# Engineering *Escherichia coli* for the production of butyl octanoate from endogenous octanoyl-CoA

**DOI:** 10.7717/peerj.6971

**Published:** 2019-07-01

**Authors:** Micaela G. Chacón, Emanuele G. Kendrick, David J. Leak

**Affiliations:** Department of Biology and Biochemistry, University of Bath, Bath, England

**Keywords:** Butyl octanoate, Alcohol acyltranferase, Metabolic engineering, Microbial production, Fatty acid metabolism

## Abstract

Medium chain esters produced from fruits and flowering plants have a number of commercial applications including use as flavour and fragrance ingredients, biofuels, and in pharmaceutical formulations. These esters are typically made via the activity of an alcohol acyl transferase (AAT) enzyme which catalyses the condensation of an alcohol and an acyl-CoA. Developing a microbial platform for medium chain ester production using AAT activity presents several obstacles, including the low product specificity of these enzymes for the desired ester and/or low endogenous substrate availability. In this study, we engineered *Escherichia coli* for the production of butyl octanoate from endogenously produced octanoyl-CoA. This was achieved through rational protein engineering of an AAT enzyme from *Actinidia chinensis* for improved octanoyl-CoA substrate specificity and metabolic engineering of *E. coli* fatty acid metabolism for increased endogenous octanoyl-CoA availability. This resulted in accumulation of 3.3 + 0.1 mg/L butyl octanoate as the sole product from *E. coli* after 48 h. This study represents a preliminary examination of the feasibility of developing *E. coli* platforms for the synthesis single medium chain esters from endogenous fatty acids.

## Introduction

Volatile short and medium chain esters (C4–C14) are found naturally in the flowering and vegetative tissue of plants where they can function either to attract pollinators, or as anti-microbial or anti-herbivore agents (Dudareva, Pichersky & Gershenzon, 2004; Beekwilder et al., 2004; Rodriguez, Tashiro & Atsumi, 2014). Short and medium chain esters also occur naturally as fermentation products from certain species of yeast and lactic acid bacteria. Those produced by the former have garnered particular interest as they strongly influence both the aroma and flavour profiles of beers and wines (Verstrepen et al., 2003; Costello et al., 2013). The majority of short and medium chain esters possess fruity and floral aromas—with their chemical structure influencing their characteristic scent. As such, they have been widely adopted as valuable flavour and fragrance compounds in perfumes, cosmetics, food, and beverages (Sose, Bansode & Rathod, 2017; Rodriguez, Tashiro & Atsumi, 2014; Schrader et al., 2004), representing a key product group in the global flavour and fragrance industry which was valued at 18.6 billion (USD) in 2015 (Grand View Research, 2017). Additionally, C4–C14 esters have a number of potential applications in the pharmaceutical, industrial solvent, and biofuel industries (Sose, Bansode & Rathod, 2017; Tai, Xiong & Zhang, 2015; Layton & Trinh, 2014; Jin et al., 2012).

Traditionally, short and medium chain esters have been obtained either through extraction from natural sources, or through chemical synthesis—the latter typically using acid and alcohol substrates derived from petroleum-based feedstocks (Welsh et al., 1989; Layton & Trinh, 2014). However, both of these approaches raise concerns about process sustainability and environmental impact (De Souza et al., 2017). An alternative ester production strategy that involves the reversal of the natural hydrolytic activity of esterases or lipases requires high levels of organic solvents (to maintain low water activity) and is less economic due to high enzyme costs (Dhake, Thakare & Bhanage, 2013; Rodriguez, Tashiro & Atsumi, 2014; Jin et al., 2012). An attractive fourth strategy for ester formation is whole-cell microbial production—whereby a host microorganism is genetically modified to produce the desired end-product(s) during growth (Layton & Trinh, 2014). Should favourable yields be achievable, microbial production offers many advantages as such processes typically use inexpensive renewable feedstocks, involve minimal quantities of hazardous reagents or side products, and can be scaled up to industrial capacity for bulk production with relative ease. As a result, the last few decades have seen the development of numerous commercial processes involving the microbial production of natural and non-natural products (Demain, 2000, 2006).

Volatile short/medium chain esters can be formed from the condensation of an alcohol and acyl-CoA catalysed by an alcohol acyl transferase (AAT). A number of AATs have been heterologously expressed, including those from *Fragaria x ananassa* (Aharoni et al., 2000; Olias et al., 1995; Horton et al., 2003); *Fragaria vesca* (Layton & Trinh, 2016; Beekwilder et al., 2004); *Clarkia breweri* (Tai, Xiong & Zhang, 2015; D’Auria, Chen & Pichersky, 2002); *Petunia hybrida* (Tai, Xiong & Zhang, 2015; Orlova et al., 2006); *Actinidia eriantha* (Layton & Trinh, 2016; Günther et al., 2011); and *Saccharomyces cerevisiae* (Rodriguez, Tashiro & Atsumi, 2014; Horton et al., 2003; Vadali et al., 2004) in heterologous hosts such as *Escherichia coli*, *S. cerevisiae*, *Clostridium acetobutylicum*, and *Lactococcus lactis* (Hernández et al., 2007; Inui et al., 2008; Park, Shaffer & Bennett, 2009; Rodriguez, Tashiro & Atsumi, 2014) to produce a variety of esters. These enzymes typically have promiscuous substrate specificity, and therefore are capable of combining multiple acyl-CoA and alcohol substrates to produce a wide range of esters (Harada, Ueda & Iwata, 1985; El-Sharkawy et al., 2005). Engineering *E. coli* to produce both the acyl-CoA and alcohol substrates endogenously for AAT mediated esterification has, for the most part, been focussed on shorter chain alcohols (ethanol, propanol, isopropanol, butanol, and isobutanol) which can be synthesized via fermentation pathways, the 2-keto acid pathway, or reverse β-oxidation (Trinh, Unrean & Srienc, 2008; Inui et al., 2008; Atsumi, Hanai & Liao, 2008; Dellomonaco et al., 2011); and shorter chain acyl-CoAs (acetyl-CoA, butyryl-CoA, and isobutyryl-CoA) which can be produced via fermentative pathways, de novo fatty acid synthesis (FAS) or reverse β-oxidation (Jawed et al., 2016; Layton & Trinh, 2014; Dellomonaco et al., 2011; Rodriguez, Tashiro & Atsumi, 2014). Additional strain improvements aimed at optimizing substrate availability for AAT catalysed esterification, by the elimination of metabolic pathways that compete directly for precursors such as pyruvate or acetyl-CoA, have succeeded in increasing final ester titres (Rodriguez, Tashiro & Atsumi, 2014; Tai, Xiong & Zhang, 2015). The culmination of these works has been particularly successful for the synthesis of short chain esters such as isobutyl acetate, isoamyl acetate, and ethyl butyrate from *E. coli* (Rodriguez, Tashiro & Atsumi, 2014; Layton & Trinh, 2016; Tai, Xiong & Zhang, 2015). However, these short chain esters are relatively toxic to the production host and, even in the presence of a typical second phase such as dodecane their amphiphilicity can still result in toxic aqueous phase concentrations.

In addition to the volatile short/medium chain esters produced via an AAT enzyme, long chain esters can be synthesized by a wax synthase (WS) enzyme. WSs have promiscuous substrate specificity, typically accepting acyl groups with chain lengths of C16 or C18 and alcohols ranging in chain length from C12 to C20 (Shi et al., 2012), though a number of WSs with activity for alcohol and acyl-CoA substrates outside of this chain length range have been characterised (Kalscheuer et al., 2006a; Shi et al., 2012; Guo, Pan & Li, 2015; Röttig, Zurek & Steinbüchel, 2015). This promiscuity has seen WS exploited for the microbial production of biodiesel, specifically fatty acid ethyl esters (FAEE) and fatty acid short esters (FASE). (Kalscheuer, Stölting & Steinbüchel, 2006b; Elbahloult & Steinbüchel, 2010; Duan et al., 2011; Zhang, Carothers & Keasling, 2012; Guo, Pan & Li, 2015; De Jong et al., 2014; Röttig, Zurek & Steinbüchel, 2015). These biodiesel strains typically produce an array of ester products where the acyl group ranges in chain length from C12 to C18 (Duan et al., 2011; Guo, Pan & Li, 2015; Röttig, Zurek & Steinbüchel, 2015). No FAEE or FASE products with an acyl chain length <C12 have been reported from biodiesel producing microbial strains. While some WS enzymes employed in these strains have been shown to accept acyl substrates with chain lengths as low as C2 in in vitro assays, the activity for C2–C12 acyl-CoAs is very low (Stöveken et al., 2005).

In contrast to the microbial production of esters composed of short chain acyl constituents (C2–C4) or long chain acyl constituents (C12–C18), the production of esters where the acyl chain length is C6–C10 has been significantly less successful, with the principal impediments being (i) the low substrate specificity of known AATs or WSs towards medium chain length acyl-CoAs, and (ii) the low endogenous availability of these medium chain length substrates. For those AAT and WS enzymes that have been characterised as having activity towards medium chain acyl-CoAs, this activity is typically low as they are often not the preferred substrate (Souleyre et al., 2005; Günther et al., 2011; Beekwilder et al., 2004; Goulet et al., 2015; Lucchetta et al., 2007; D’Auria, Chen & Pichersky, 2002). This proves especially problematic in an intracellular environment as the milieu is highly crowded and contains a wide range of potential substrates, increasing the likelihood of medium chain acyl-CoAs being outcompeted by others that bind at higher affinity to the AAT or WS (Souleyre et al., 2005; Layton & Trinh, 2016; Verstrepen et al., 2003). Since medium chain acyl-CoAs are naturally present only at low abundances in *E. coli*, generally being found as intermediates of β-oxidation (Torella et al., 2013; Lennen & Pfleger, 2012), the background of preferred substrates becomes more problematic. However, recent success has been achieved in the engineering of *E. coli* for the increased production of free medium chain acids (Torella et al., 2013; Clomburg et al., 2015; Wu et al., 2017; Tan et al., 2018), making the possibility of producing high titres of medium chain fatty acid derivatives (via an acyl-CoA intermediate), such as esters, more feasible.

The present proof-of-concept study describes the construction of an *E. coli* strain for the production of a single medium acyl-chain ester, butyl octanoate, from endogenous octanoyl-CoA. While butyl octanoate has widespread commercial applications, it also represents a molecule with attractive production characteristic. Unlike shorter chain esters it is non-toxic to *E. coli* (Fig. S1) while also sufficiently permeable to diffuse through the cell membrane, unlike longer chain esters. Therefore, if a good production strain could be generated it offers the potential to generate a product which could naturally accumulate as a non-toxic second phase. The microbial production of this compound has not been extensively explored to date, due to obstacles such as those described above (Sose, Bansode & Rathod, 2017), while the majority of work describing ester synthesis in *E. coli* has reported the production of ester mixtures (Rodriguez, Tashiro & Atsumi, 2014; Layton & Trinh, 2014; Guo, Pan & Li, 2015)—most likely a result of the combination of AAT/WS promiscuity and the endogenous availability of multiple potential substrates. Downstream separation of these mixtures by fractional distillation would increase process cost (Onuki et al., 2008). Therefore, in order to capitalise on both the potential for self-partition and to minimise downstream purification, it was desirable to engineer *E. coli* for single ester production. In this work, butyl octanoate was obtained as a single end-product from *E. coli* by engineering an AAT enzyme from *Actinidia chinensis* for improved product specificity for butyl octanoate, and incorporating it into a strain of *E. coli* modified for high octanoyl-CoA availability. This is the first example of a medium chain ester with an acyl chain >C6 being produced as a single ester product in *E. coli*.

## Materials and Methods

All media components, solvents and chemicals were purchased from Sigma-Aldrich (Dorset, UK) or Fisher Scientific (Loughborough, UK) unless otherwise stated. *E. coli* strains BIO*Blue* (Bioline, London, UK), C43 (DE3) (Lucigen, Middleton, WI, USA), and S002 (Torella et al., 2013) were used for plasmid construction and expression, respectively. Gene sequence harmonisation was performed manually using an *Actinidia chinensis* codon usage table created using data analysis and molecular biology and evolution, (Xia, 2013) software, while all gene sequence optimisation and gene synthesis was performed by GeneArt (Thermo Fisher Scientific, Waltham, MA, USA). Plasmid pBEST-luc was purchased from Addgene as plasmid #45394 (a gift from Vincent Noireaux), as was plasmid pAG32 (#35122; a gift from John McCusker). *E. coli* strain S002 was a generous gift from Pamela A. Silver (Harvard Medical School).

### Plasmids, bacterial strains, and growth conditions

All plasmids and strains used in this study are listed in Table 1. Luria Broth (LB) media (10 g/L tryptone, 10 g/L NaCl, and 5 g/L yeast extract) was used for gene cloning procedures and pre-culturing. For shake-flask production of butyl esters, recombinant strains were cultured either in Terrific Broth (TB) media (12 g/L tryptone, 24 g/L yeast extract, 4 mL glycerol, 0.17 M KH_2_PO_4_, and 0.72 M K_2_HPO_4_) containing 2% glucose (w/v), or LB media (10 g/L tryptone, 5 g/L yeast extract, 10 g/L NaCl) containing 2% glucose (w/v). For tube production of octanoic acid, recombinant strains were cultured in M9 media (33.9 g/L Na_2_HPO_4_, 15 g/L KH_2_PO_4_, 5 g/L NH_4_Cl, 2.5 g/L NaCl pH 7.4, 2 mM Mg_2_SO_4_, 0.1 mM CaCl_2_, 3 nM (NH_4_)6Mo_7_O_24_·4H_2_O, 0.4 μM H_3_BO_3_, 30 nM CoCl_2_·6H_2_O, 10 nM CuSO_4_·5H_2_O, 80 nM MnCl_2_·4H_2_O, 10 nM ZnSO_4_·7H_2_O, 1 μM FeSO_4_·7H_2_O) containing 10 g/L glycerol (v/v). Ampicillin (Amp, 100 μg/mL) or hygromycin B (HygB, 100 μg/mL) were added to the culture medium depending on the selectable marker gene on each plasmid.

**Table 1:** Description of *E. coli* plasmids and strains used in this study.

Name	Description	Reference
Plasmid		
pET21a::AAT16	pColE1, Amp^R^, T7, *AAT16*_Ac_	This study
pET21a::AAT16-S99G	pColE1, Amp^R^, T7, *AAT16-S99G*_Ac_	This study
pET21a::AAT16-L178F	pColE1, Amp^R^, T7, *AAT16-L178F*_Ac_	This study
pET21a::AAT16-F185I	pColE1, Amp^R^, T7, *AAT16-F185F*_Ac_	This study
pET21a::AAT16-S99G-L178F	pColE1, Amp^R^, T7, *AAT16-S99G-L178F*_Ac_	This study
pRSETa::FATB1	pColE1, Amp^R^, T7, *FATB1*_Cp_	This study
pBEST-Luc	p15A, Amp^R^, P_L_lacO_l_, *GFP*	Shin & Noireaux (2010)
pAG32	pBR322, Amp^R^, T7, *HygB^R^*	Goldstein & McCusker (1999)
pBEST03	p15A, HygB^R^, T7, *AAT16-S99G*_Ac_, T7, *FATB1*_Cp_, *FadD-V451A*_Ec_	This study
Strains		
BIO*Blue*	*rec*A1, *end*A1 *gyr*A96 *thi*-1 *hsd*R17(r_k_-, m_k_+) *sup*E44 *rel*A1 *lac* [F′ *pro*AB *lac*I^q^ZΔM15 *Tn*10(Tet^r^)]	Bioline
C43 (DE3)	F—*ompT hsdSB (rB-mB-) gal dcm* (DE3)	Lucigen
S002	BL21 (DE3) *ΔfadE*	Torella et al. (2013)
DLBO1	S002 harbouring pET21a::*AAT16*	This study
DLBO2	S002 harbouring pET21a::*AAT16-S99G*	This study
DLBO3	S002 harbouring pET21a::*AAT16-S99G* and pBEST03	This study
DLBO5	S002 harbouring pRSETa::*FATB1*	This study

**Notes:**

*E. coli* plasmids and strains used in this study.

In the plasmid description, subscript letters refer to the source of the gene as follows: Ac, *Actinidia chinensis*; Cp, *Cuphea palustris*; Ec, *Escherichia coli*.

### Plasmid construction

All plasmids constructed were sequenced for verification by GATC Biotech (Konstanz, Germany). All primer sequences can be found in Table S1. The harmonised coding sequence of *AAT16* from *Actinidia chinensis* (GenBank No. HO772640; harmonised sequence in Fig. S2) was cloned between restriction sites *Bam*HI and *Xho*I into the IPTG-inducible *E. coli* expression vector pET21a to produce pET21a::*AAT16*. Derivative point mutants of the *AAT16* gene were made by overlap extension PCR consisting of two sequential DNA amplifications, as described by Heckman & Pease (2007). The gene sequence for each AAT point mutant was flanked with *Bam*HI and *Xho*I restriction sites and inserted into pET21a. The optimised coding sequence of *FATB1* from *Cuphea palustris* (GenBank No. AAC49179.1; optimised sequence in Fig. S2) was cloned between restriction sites *Xho*I and *Nco*I into the IPTG-inducible *E. coli* expression vector pRSETa to produce pRSETa::*FATB1*. A description of how plasmid pBEST03—containing the harmonised *AAT16-S99G* point mutant from *Actinidia chinensis*, optimised *FATB1* from *Cuphea palustris*, and optimised *FadD-V451A* point mutant from *E. coli*—was created from the template plasmids: pET21a::*AAT16-S99G*, pBEST-Luc, and pAG32, can be found both in the Supplementary Methods and Fig. S5.

### Identification of amino acid residues for mutagenesis

#### 3D protein structure modelling

A 3D model of AAT16 from *Actinidia chinensis* was built by comparative modelling based on the high-resolution crystal structure of homologous proteins. Swiss-Model (Arnold et al., 2006) was used for selecting protein structures with closest homology to AAT16 available in the PMDB. The crystal structures of three proteins (PMDB codes: 4KEC, 4G22, and BGH2) were selected as the template for AAT16 model construction, which was then done using MODELLER 9.18 software (www.salilab.org/modeller/). Five models were selected based on their discrete optimized protein energy score and their root mean squared deviation (RMSD) with respect to trace (Cα) atoms of the crystal structure templates. A loop optimisation protocol was then used to improve the quality of each model. Finally, MolProbity and VERIFY3D (Chen et al., 2010; Lüthy, Bowie & Eisenberg, 1992) were employed to evaluate the accuracy of the models, and the one with the best stereochemistry was chosen.

#### Binding pocket identification

The solvent pocket associated with the AAT16 active site motif was identified using the ICMPOCKETFINDER software, which uses a transformation of the Lennard-Jones potential to predict the position and size of ligand-binding pockets (Abagyan, Totrov & Kuznetsov, 1994; www.molsoft.com).This feature identifies all voids and pockets of the protein 3D structure and measures the volume and area of each. As well, it identifies the surrounding residues that compose said pockets. The pocket associated with the acyltransferase catalytic motif (HxxxD) was identified as the substrate binding pocket and is found to be highly conserved in higher plants and yeast (Morales-Quintana et al., 2011).

#### Molecular docking of AAT mutants

Docking studies were carried out to predict putative modification in the binding mode of butanol of octanoyl-CoA between the wild type AAT16 protein and mutant variants of the AAT16 protein. This was carried out using ICM software (www.molsoft.com). First, a library of AAT16 mutants was created where those residues that influence the substrate binding pocket (identified in section “Binding pocket identification”) were individually mutated to the corresponding residue(s) present at that location in other AAT enzymes. This was done as an attempt to prevent wholly unfavourable mutations with the presumption that should a given residue be present at that location in another AAT, that a mutation to that residue in the AAT16 protein is less likely to result in complete abolishment of activity. Where more than one residue was present at a given location in different AAT enzymes, each permutation was included. This library was then used for docking investigations whereby the octanoyl-CoA was bound first, and the butanol second. Resulting docking conformations were then assessed and optimized as described by Galaz et al., 2013. The orientation and distance of the docked substrates to the catalytic residues present in the active site were then measured and compared to the wild type AAT16.

### Shake flask characterisation of butyl ester production in *E. coli*

#### Expression of AAT16 from Actinidia chinensis with exogenous butanol and octanoic acid feeding

*Escherichia coli* strain C43 (DE3) harbouring pET21a::*AAT16* was grown in LB at 37 °C and 250 rpm, overnight. Cultures were inoculated to 1% (v/v) in 25 mL of TB + 20 g/L glucose and grown at 37 °C and 250 rpm until on OD_600_ of 0.8–1.0 was reached. Cultures were then induced by the addition of 0.4 mM IPTG before being transferred to a rotary shaker (250 rpm) at 20 °C for 2 h. Cultures were then supplemented with 10 mM butanol and five mM octanoic acid before being returned to 250 rpm and 20 °C for a further 18 h. Following this, culture flasks were rested at −4 °C for 30 min to condense volatilized esters before the OD_600_ of each culture was determined and then centrifuged at 4,000 rpm for 15 min the separate the pellet and supernatant fractions, the supernatant fraction was extracted directly into hexane, while the pellet fraction was resuspended in five mL of 0.9% NaCl (w/v) and sonicated at 12 microns for 30 s before being extracted into hexane for product analysis by Gas Chromatography-Mass Spectometry (GC-MS).

For the comparison of the butyl ester profile between wild type AAT16 and AAT16 point mutants, *E. coli* strain C43 (DE3) harbouring pET21a::*AAT16*; *AAT16-S99G*; *AAT16-L178F*; or *AAT16-S99G-L178F* were cultured as described above in a total volume of 5 mL in glass test tubes.

#### Expression of strain DLBO3 with exogenous butanol feeding

*Escherichia coli* strain DLBO1 (control) and DLBO3 were grown in LB at 37 °C and 250 rpm, overnight. Cultures were inoculated to 1% in 25 mL of TB + 20 g/L glucose (w/v) and grown at 37 °C and 250 rpm until on OD_600_ of 1.0 was reached. Cultures were then induced by the addition of 0.4 mM IPTG and supplemented with 4 μg/mL cerulenin before being transferred to a rotary shaker at 20 °C for 2 h. Cultures were then supplemented with 10 mM butanol before being returned to 250 rpm and 20 °C for a further 24 or 48 h. Following this, the OD_600_ of each culture was determined and sample preparation for analysis by GC-MS was carried out as described above in section ‘Expression of AAT16 from *Actinidia chinensis* with exogenous butanol and octanoic acid feeding’.

### Alcohol acyl transferase activity assay

*Escherichia coli* strain C43 (DE3) containing either no plasmid (negative control), pET21a::*AAT16*, pET21a::*AAT16-S99G*, or pET21a::*AAT16-L178F* were grown at 37 °C in LB overnight. Cultures were then inoculated to 1% in 250 mL of TB +/− ampicillin (100 μg/mL) and grown at 37 °C and 250 rpm until an OD_600_ of 1.0 was reached. At this point gene expression was induced with the addition of 0.4 mM IPTG and cultures were further incubated another 16 h at 18 °C. Following this, *E. coli* cells were harvested through centrifugation (4,000 rpm for 40 min), resuspended in extraction buffer (50 mM Tris HCl pH 8.5, 10% (v/v) glycerol, and 1 mM DTT; Complete™ protease inhibitor tablet-EDTA-free, Roche, Basel, Switzerland), and sonicated on ice until clear. Samples were then centrifuged (4,000 rpm for 20 min) to pellet cell debris, and the crude cell lysates were concentrated using Amicon 10 kDa MWCO centrifugal filters. Protein concentration was determined using the Bradford assay (Bradford, 1976).

Reactions were carried out in a total volume of 500 μL containing: 20 μL crude cell lysate of *E. coli* strain C43 (DE3) expressing either AAT16, AAT16-S99G, or AAT16-L178F enzyme, buffer (50 mM Tris HCl pH 8.5, 10% (v/v) glycerol, and one mM DTT), 20 mM butanol, and 0.5 mM octanoyl-CoA. Both substrates were present at saturating concentrations. Reactions were incubated at 30 °C and 250 rpm for 30 min before being halted with the addition of 100 μL of 10% (w/v) SDS. Each reaction was then extracted into 150 μL of hexane for future product analysis by GC-MS. All assays were carried out in triplicate.

### Endogenous octanoic acid production analysis

*Escherichia coli* strains C43 (DE3), S002, and DLBO2 were grown at 37 °C in LB overnight. Cultures were then inoculated to 1% in 7 mL of M9 media + 1% glycerol (v/v) (+/− 100 μg/mL ampicillin) and grown at 37 °C and 250 rpm until an OD_600_ of 0.8, and then induced with 0.4 mM IPTG. At this time, some cultures were additionally supplemented with 4 μg/mL of the antibiotic cerulenin for fatty acid elongation retardation. Following induction, all cultures were incubated at 30 °C and 250 rpm for 24 h before analysis. For analysis, the OD_600_ of each culture was determined, and 2 mL of culture was centrifuged at 4,000 rpm for 10 min to isolate the cell pellet. 10 μg of undecanoic acid (C:11) was added as an internal standard to the cell pellet before resuspension in three mL of 1 M MeOH-HCl. Samples were then incubated at 80 °C for 90 min for lipid transmethylation. Samples were then cooled to room temperature before the addition of 1 mL of 0.9% (w/v) NaCl. Following this, each sample was extracted into hexane for methyl ester analysis by GC-MS.

### Ester and acid quantification

Butyl ester products were quantified using a model 7890B gas chromatograph and 5977A mass spectrometer (Agilent technologies, Stockport, UK). Samples were separated on a DB-FFAP 30m × 20 μm × 0.25 μm capillary column under the following conditions: one μL of sample was injected onto the column which was held at 40 °C for 5 min, the temperature was then ramped at a gradient of 15 °C/min to a final temperature of 250 °C and held for 4 min. Butanol and butyl ester products typically eluted between 4 and 12 min and were monitored on both MS and FID detectors. The concentration of product was quantified using calibration curves for each compound analysed.

## Results and Discussion

### Expression of AAT16 from *Actinidia chinensis* in *E. coli*

**Figure 1:**
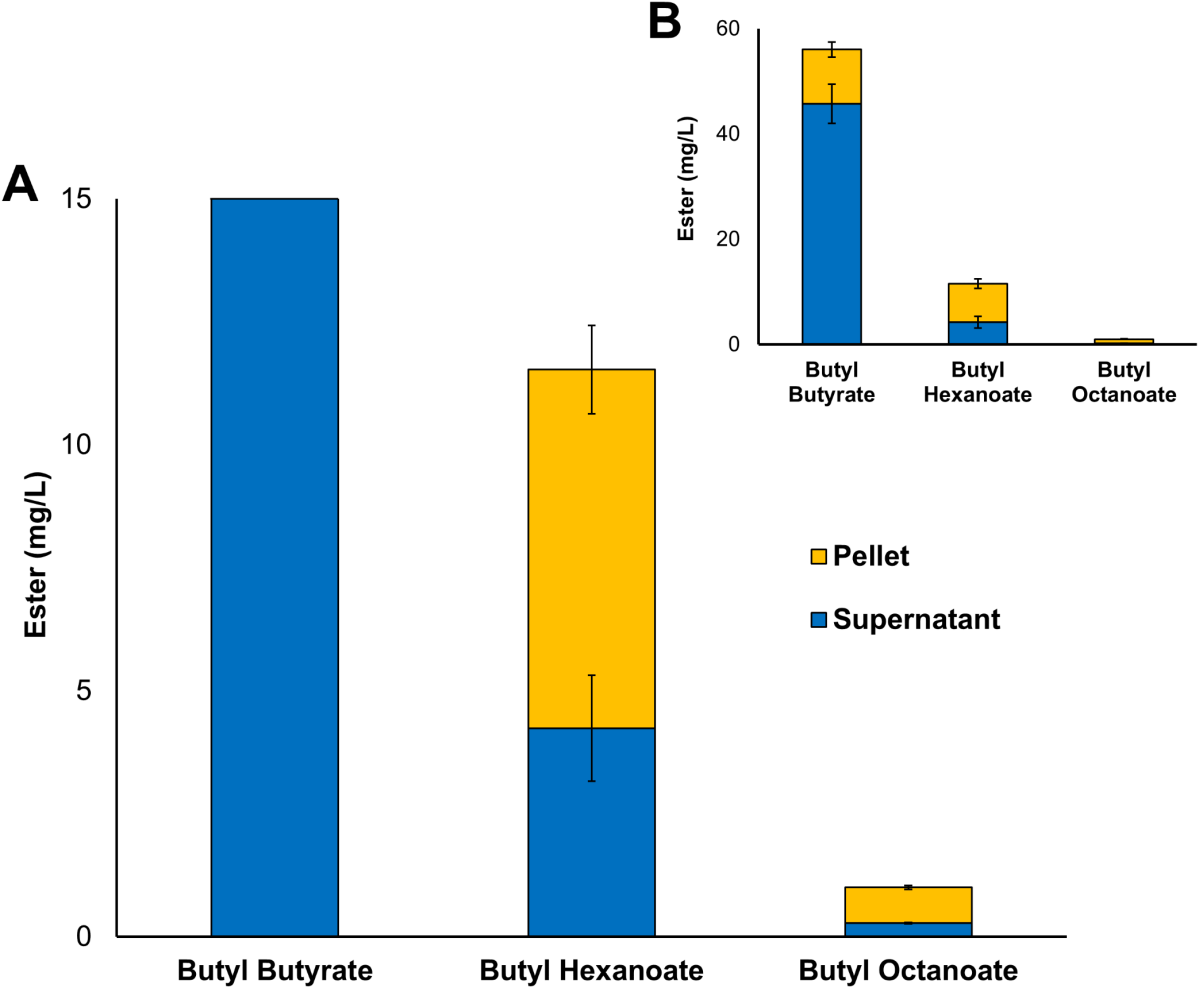
Total production of butyl butyrate, - hexanoate, and -octanoate from *E. coli* C43 (DE3) expressing the codon harmonised AAT16 from *A. Chinensis* upon exogenous addition of 10 mM butanol and 5 mM octanoic acid. (A) shows the proportion of butyl octanoate present in the supernatant and pellet fractions, while (B) shows the proportion of each butyl ester produced. Products were analysed from both the culture supernatant and cell pellet after 18 h of growth at 20 °C. Final titres are based on culture volume. Data are the mean ± standard deviation from three biological replicates.

Three alcohol acyltransferase enzymes have previously been reported as being able to catalyse the formation of butyl octanoate, these are: AeAAT9 from *Actinidia eriantha*, AAT16 from *Actinidia chinensis*, and MpAAT1 from *Malus pumila* (Souleyre et al., 2005; Günther et al., 2011). An AAT purified from *Neurospora sp*. ATCC 46892 has also shown activity towards both butanol and octanoyl-CoA in vitro, however, to date no *Neurospora* AAT coding gene has not been assigned (Yamauchi et al., 1989; Menendez-Bravo et al., 2017). While butyl octanoate is not the preferred end-product for any of these three enzymes, AAT16 from *Actinidia chinensis* showed the highest activity for its production, and so was chosen as the candidate AAT for this work (Günther et al., 2011). No WS enzymes were considered in this study as none have been shown to produce butyl octanoate either in vitro or in vivo. The nucleotide sequence of the *AAT16* gene was codon-harmonized for expression in *E. coli* and cloned into pET21a to drive expression from the strong T7 promoter (Table 1). For initial studies, expression cultures of *E. coli* C43 (DE3) harbouring the pET21a::*AAT16* plasmid were supplemented with 10 mM butanol and 5 mM octanoic acid to provide an excess of substrate to the AAT16 enzyme. It was found that these cultures produced a mixture of acyl butyl esters that included butyl butyrate, -hexanoate, and -octanoate after 18 h incubation (Fig. 1). Butyl esters were not detected in *E. coli* cultures expressing AAT16 with no substrate supplementation. These esters were found both in the culture broth and cell pellet, with the proportion of ester found in the cell pellet fraction increasing as the chain length of the ester increased (Fig. 1). This probably reflects the increased hydrophobicity of the longer octanoic ester compared to the more hydrophilic butyric ester, promoting its partition into the cell membrane. The butyl butyrate (54 + 5 mg/L) and -hexanoate (11 + 2 mg/L) titres were significantly higher than that of butyl octanoate (1 + 0.06 mg/L). This is a reflection of the substrate specificity of the AAT16 enzyme, which prefers the shorter butyryl-CoA and hexanoyl-CoA chain lengths to octanoyl-CoA (Günther et al., 2011). Cultures of *E. coli* C43 (DE3) harbouring pET21a::*AAT16* supplemented with only butanol produced a small amount of butyl butyrate and -hexanoate, but no butyl octanoate (Fig. S8). This suggests that a portion of the butanoyl-CoA and hexanoyl-CoA substrate utilized by AAT16 was being derived from endogenous acyl-CoA pools and/or fatty acids taken up from the culture broth.

The transport of a free fatty acid across the cytoplasmic membrane of *E. coli* is coupled with its esterification to an acyl-CoA. In this active form it can then act as a substrate for phospholipid biosynthesis or β-oxidation (Zhang, Wang & Qi, 2006; Janßen & Steinbüchel, 2014; Yao & Rock, 2015). The results of Fig. 1 suggest that upon its activation to octanoyl-CoA, a significant proportion of the exogenously supplied octanoic acid was entering the β-oxidation pathway where it was being iteratively oxidized to hexanoyl-CoA and butanoyl-CoA—substrates that AAT16 was then acting on preferentially. These observations suggest that engineering an *E. coli* system for the production of butyl octanoate as the sole product would benefit from protein engineering of the AAT16 enzyme for improved activity towards the longer octanoyl-CoA chain length in order to prevent the esterification from being a rate limiting step and also, removal of β-oxidation.

### Protein engineering of AAT16 for improved activity towards octanoyl-CoA

#### Homology modelling of AAT16 and in silico mutant analysis

**Figure 2:**
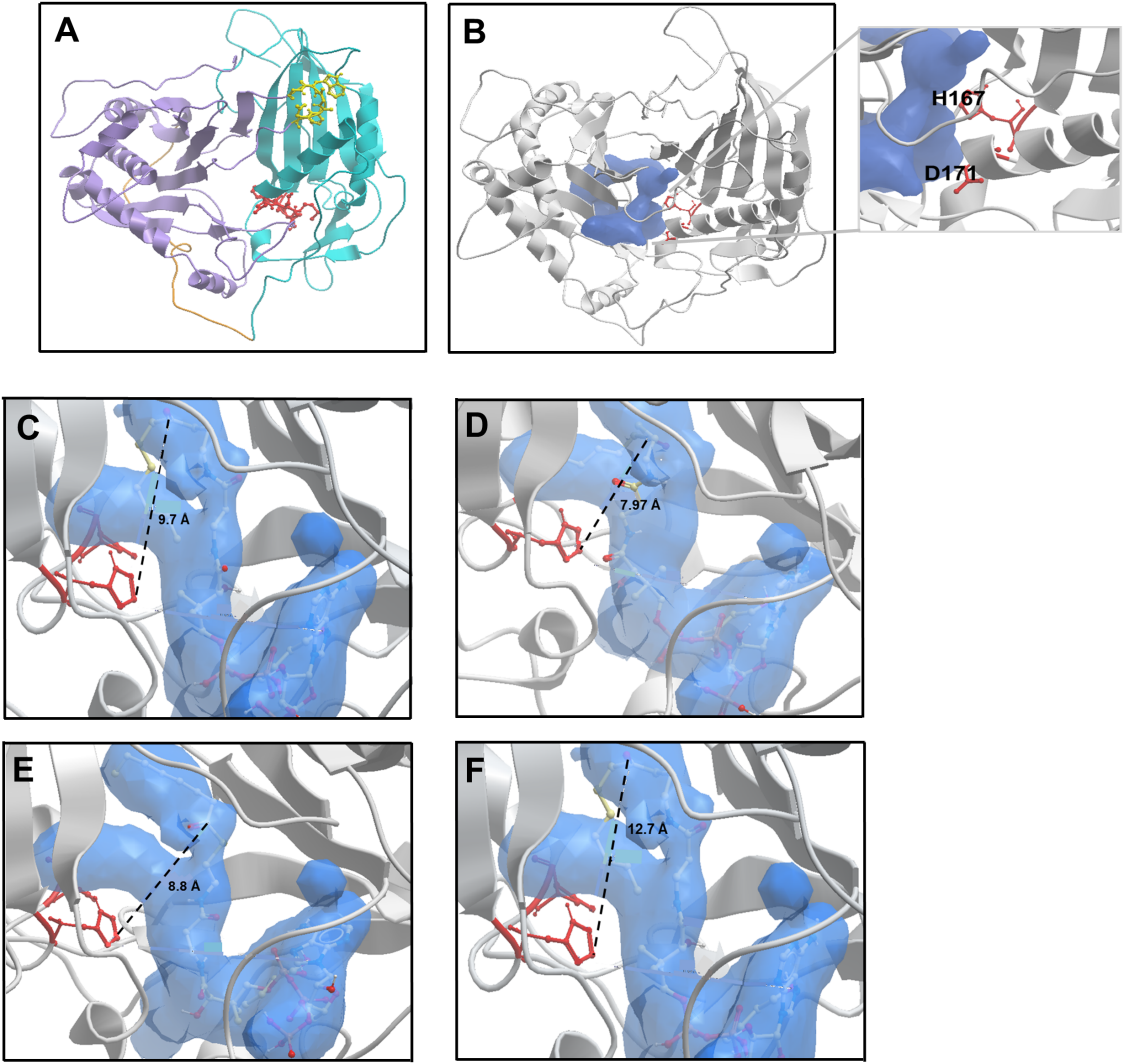
Structural model of the AAT16 protein from *A. chinensis*, and the docking of butanol and octanoyl-CoA within the active site of AAT16-wt, AAT16-S99G, AAT16-L178F, and AAT16-F185I. (A) Structural model of the AAT16 protein from *A. chinensis* created with Modeller 9.18 software. Domain I = blue, cross over loop = orange, domain II = purple, HXXXD catalytic motif = yellow, and DFGWG structural motif = red. (B) Predicted substrate binding pocket (shown in blue) in relation to the whole protein and catalytic residues (red). Inset shows a close-up of the solvent channel and its proximity to residues H167 and D171. (C–E) Spatial distribution of substrates butanol and octanoyl-CoA in the active site of each protein-ligand model. A detailed view of the active site of: wild type AAT16 (C); AAT16-S99G (D); AAT16-L178F (E); and AAT16-F185I (F). Distance (Å) from the ɛ nitrogen of H167 to the carbonyl group of the octanoyl-CoA and the hydroxyl group of the butanol is indicated.

Alcohol acyltransferase proteins are members of the BAHD superfamily (BAHD is an acronym made of the initial letters of the first four enzymes characterised as belonging to this family). The first member of the acyl-CoA dependent BAHD acyl transferase superfamily to be crystallised was a vinorine synthase from *Rauvolfia serpentine*, and from this structure it was determined that members of the BAHD family are composed of two equal sized domains connected via a large crossover loop (Ma et al., 2005). Additionally, they share two conserved motifs: an HxxxD catalytic motif located in the middle of the protein sequence, and a C-terminal DFGWG motif involved in maintaining protein structural integrity (Galaz et al., 2013; Morales-Quintana et al., 2013). With no available crystal structure for AAT16 from *Actinidia chinensis*, a model was constructed using three protein templates: a hydroxycinnamoyl CoA:shikimate hydroxycinnamoyl transferase from Sorghum (PDB: 4KE4); a hydroxycinnamoyl-CoA shikimate/quinate hydroxycinnamoyl transferase from *Coffea canephora* (PDB: 4G22), and the vinorine synthase from *R. serpentine* (PDB: 2BGH) (Ma et al., 2005; Lallemand et al., 2012; Walker et al., 2013). These templates had sequence identities of 28.2%, 32.9%, and 33.3% to AAT16, respectively. Of the models generated, the best AAT16 model conformer was then further refined and validated based on stereochemical quality using MolProbity software (Chen et al., 2010). From this it was found that 99.3% of amino acid residues were in favourable or allowed regions (Fig. S3). The final AAT16 protein model can be seen in Fig. 2A where domain I, domain II, the crossover loop, the HxxxD motif, and the DFGWG motif are highlighted. Previously, several groups have constructed protein models for AAT enzymes from melon (*Cucumis melo*), mountain papaya fruit (*Vasconcellea pubescens*), and strawberry (*Fragaria x ananassa*) and paired in silico and in vitro analysis to characterise the mechanism of catalysis for these enzymes, as well as the impact of AAT structure on substrate specificity (Morales-Quintana et al., 2011, 2013; Galaz et al., 2013; Navarro-Retamal et al., 2016). From this work, it has been determined that both the alcohol and acyl-CoA substrates access the catalytic His and Asp residues of the HxxxD motif through a solvent channel that runs through the centre of the protein between domains I and II. Consistent with this, the AAT16 model possesses a predicted central cavity into which H167 and D170 are exposed to facilitate interaction with alcohols and acyl-CoAs (Fig. 2B and inset). This cavity in AAT16 has an area of 903.8 Å^2^ and a volume of 973.7 Å^3^. The proposed mode of AAT catalysis involves the formation of a ternary complex between the acyl-CoA, alcohol, and protein, which is consistent with the mechanism of catalysis described for other BAHD family members such as: Ss5MaT1, CAT and HAT—for which the mechanistic features of acyl transfer have been extensively studied (Leslie, 1990; Lewendon et al., 1994; Tanner et al., 1999; Suzuki, Nakayama & Nishino, 2003; Galaz et al., 2013). Molecular dynamics simulations of VpAAT1 from *V. pubescens* suggest that the acyl-CoA substrate interacts with the His residue of the HxxxD motif while the alcohol substrate initially interacts with Asp. The alcohol then reorients towards the His via intermediate interaction with a serine residue to eventually produce an acyl-CoA-alcohol-His complex (Morales-Quintana et al., 2013). Here, the His residue can deprotonate the hydroxyl group of the alcohol to facilitate its nucleophilic attack on the carbonyl group of the acyl-CoA. Molecular dynamics simulations have shown that throughout catalysis the distance between the acyl-CoA substrate and the His residue remains relatively unchanged (Morales-Quintana et al., 2013; Galaz et al., 2013). Ligand binding analysis of the AAT16 model with octanoyl-CoA was performed using in silico substrate docking in order to analyse the orientation of octanoyl-CoA to the catalytic H166 residue. Ternary complexes were obtained by first building the octanoyl-CoA-AAT16 complex and then incorporating the butanol. It was found that the octanoyl-CoA was located 9.7 Å from H166, which is an unfavourable distance and may explain the low activity of AAT16 for making octanoyl esters (Fig. 2C). As it has previously been determined that the structure of the solvent channel of AAT is crucial for activity and substrate specificity (Morales-Quintana et al., 2011; Galaz et al., 2013), we hypothesized that certain amino acid mutations may alter the orientation of the octanoyl-CoA substrate in the solvent channel, improving its position with regards to H166. To investigate this a number of *in silico* mutants were generated and evaluated for substrate docking. Two of these mutants were selected for experimental investigation: S99G and L178F as they both decreased the distance between the carbonyl group of octanoyl-CoA and H166 in the binding pocket. Additionally, an F185I mutant, which resulted in an increased distance between octanoyl-CoA and the catalytic base (in silico), was selected for comparison. Modelling of these mutations suggested that none would have a significant impact on the pocket shape, orientation of the catalytic residues, or protein backbone compared to AAT16-wt. The AAT16-wt structure was superimposed on each of the mutants, and in each case the proteins displayed similar 3D structures as well as an RMSD value of the backbone of the whole structures that suggested good overall alignment (Fig. S4). Interestingly, amino acid alignments of AAT16 with other fruit or flower derived AAT enzymes from different species showed that the amino acids at positions 99, 178 and 185 were relatively well conserved among the other AATs, with AAT16 having divergent residues at these positions (Fig. S5). For each of these three mutants, the original residue was changed to the most common residue found at this position among other AATs. It was found that the solvent channels of each mutant remained structurally similar to AAT16-wt, though the pocket volume of AAT16-S99G and AAT16-L178F increased (982.4 and 979.3 Å^3^, respectively) while the pocket volume of AAT16-F185I decreased (969.4 Å^3^). Substrate docking showed that the distance between octanoyl-CoA and H166 was reduced for both AAT16-S99G and AAT16-L178F to 7.97 and 8.8 Å, respectively, while it increased for AAT16-F185I to 12.7 Å (Figs. 2C–2F).

#### In vivo and in vitro analysis of AAT16 mutants

**Figure 3:**
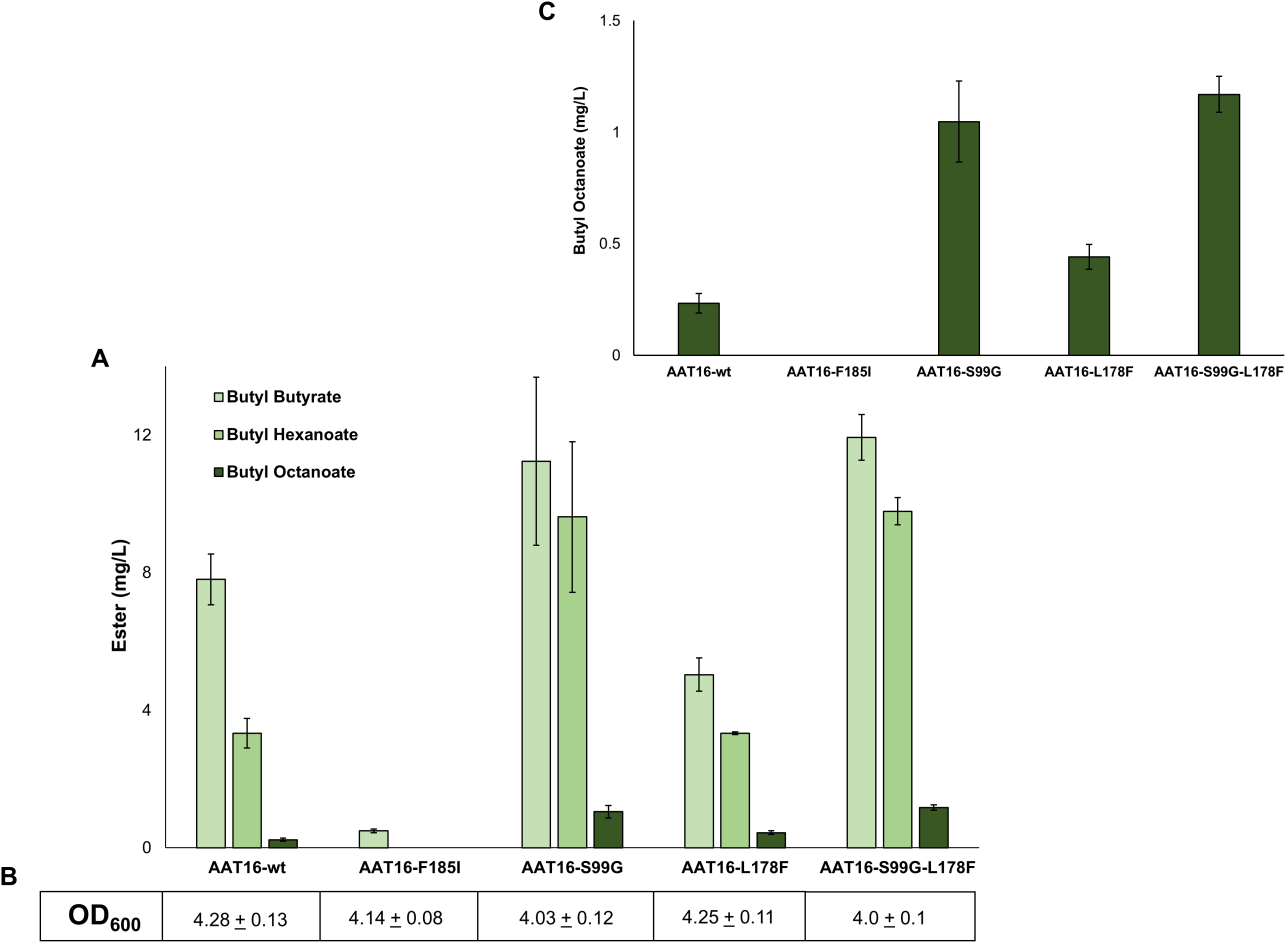
(A) Proportion of butyl butyrate, -hexanoate, and -octanoate present in the culture broth of *E. coli* C43 (DE3) expressing either the AAT16, AAT16-S99G, AAT16-L178F, or AAT16-S99G-L178F upon exogenous addition of 10 mM butanol and 5 mM octanoic acid. Products were analysed after 18 h of growth at 20 °C. (B) shows the final culture OD_600_. The inset above (C) shows the proportion of butyl octanoate produced by each of these four proteins. Data are the mean ± standard deviation of three biological replicates.

To investigate the impact of each of these mutations on butyl octanoate production in vivo, each AAT16 mutant was created using overlap PCR and cloned into pET21a. Expression cultures of *E. coli* C43 (DE3) harbouring either pET21a::*AAT16-wt*, *AAT16-F185I*, *AAT16-S99G*, or *AAT16-L178F* were supplemented with 10 mM butanol and 5 mM octanoic acid and the butyl ester profile from the culture broth was analysed after an 18 h incubation. As predicted, the AAT16-F185I mutant had significantly reduced ester production compared to AAT16-wt (Fig. 3). While hexanoyl-CoA and butanoyl-CoA docking was not assessed *in silico*, this may also be the case for these substrates as well. In contrast, the AAT16-S99G and AAT16-L178F mutants respectively produced 4.5 times and 1.9 times more butyl octanoate compared to AAT16-wt (Fig. 3 and inset). Consistent with predictions from modelling the S99G mutant, for which the octanoyl-CoA was predicted to dock closest to the H166 residue, resulted in the largest improvement in butyl octanoate titres. Interestingly, the S99G mutant appeared to produce significantly more butyl hexanoate compared to the AAT16-wt, while the L178F mutant produced significantly less butyl butyrate (Fig. 3). This suggests that it may be possible not only to open the active site to accommodate larger fatty acyl-CoAs but also to selectively reduce activity towards the shorter chain homologues. A double mutant, AAT16-S99G-L178F, was created and assessed *in vivo* to determine if together these mutations could further increase butyl octanoate accumulation in *E. coli*. However, no significant improvement in titre was observed from the double mutant compared to the AAT16-S99G single mutant, and the proportion of each ester produced was similar to this single mutant as well (Fig. 3). This was consistent with *in silico* substrate docking, which found that octanoyl-CoA was not predicted to dock closer to H166 in the solvent channel of AAT16-S99G-L178F than in AAT16-S99G (Fig. S12). As the extent of growth (Fig. 3) of cultures of *E. coli* harbouring plasmids of AAT-wt and each variant and the AAT protein abundance (determined by SDS-PAGE densitometry, Fig. S10) were found to be similar this suggests that the final butyl ester titres and distribution of esters observed *in vivo* were a genuine reflection of enzyme substrate specificity. To further confirm this, the catalytic activity of AAT16-wt, AAT-S99G, AAT-L178F, and AAT16-S99G-L178F for making butyl octanoate was assessed by performing *in vitro* assays using crude cell extract (Table 2). It was found that each mutant had improved activity for producing butyl octanoate over the AAT16-wt, and that the activities seen *in vitro* correlated well with the results seen *in vivo* (Fig. 3).

**Table 2:** *In vitro* activity of the AAT16 enzyme and its single point mutants for making butyl octanoate.

Enzyme	Activity (nmol/min/mg total protein)
No plasmid^a^	ND
AAT16-wt	1.1 ± 0.3
AAT16-S99G	4.4 ± 0.4
AAT16-L178F	2.0 ± 0.3
AAT16-S99G-L178F	4.5 ± 0.2

**Notes:**

Data represents the average of three replicates ± standard deviation.

Assays were performed at 30 °C for 30 min using 20 mM butanol and 0.5 mM octanoyl-CoA.

a C43(DE3) carrying no plasmid was used as a negative control.

While the S99G and L178F mutants of AAT16 resulted in increased butyl octanoate accumulation upon heterologous expression and substrate feeding in *E. coli*, final titres remained low. The continued high production of butyl butyrate and -hexanoate from the mutants showed that these shorter acyl chain lengths remained the preferred substrates. While additional AAT16 protein engineering could be performed to further improve the activity of this enzyme towards octanoyl-CoA, it is unlikely to lead to an absolute specificity for this substrate in the presence of shorter chain acyl-CoAs. Therefore, to increase butyl octanoate production it was necessary to reduce short chain acyl-CoA production from octanoyl-CoA by perturbation of the β-oxidation pathway (Steen et al., 2010; Duan et al., 2011; Layton & Trinh, 2014).

### β-oxidation perturbation for improved butyl octanoate product specificity from AAT16 in *E. coli*

**Figure 4:**
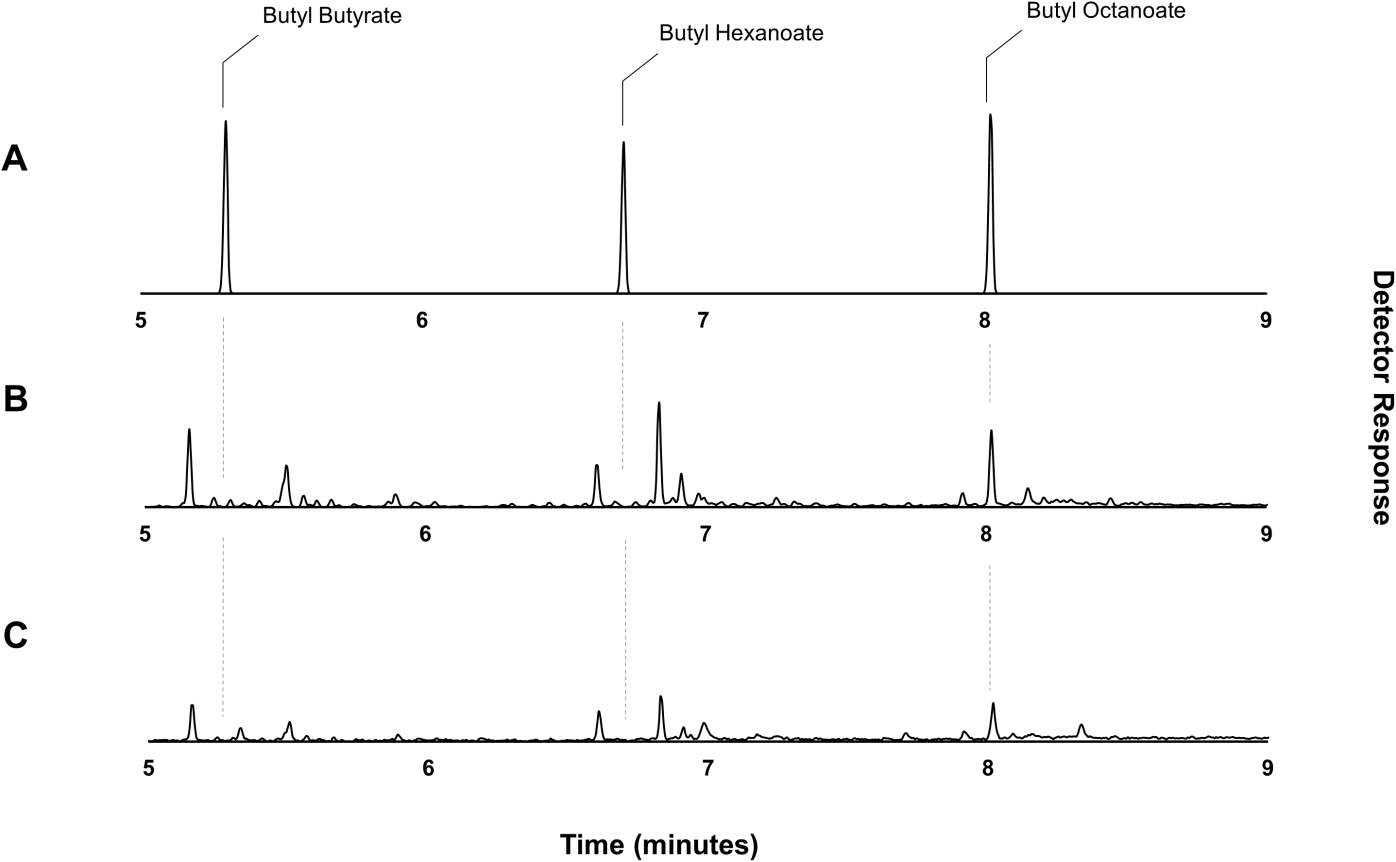
Gas chromatograms showing butyl octanoate production from strain DLBO1. Gas chromatograms showing butyl ester production of strain DLBO1 supplemented with 10 mM butanol and 5 mM octanoic acid after 18 h of growth at 20 °C. (A) shows the retention times of butyl butyrate, -hexanoate, and -octanoate standards, (B) shows the butyl ester profile of the cell pellet, and (C) shows the butyl ester profile of the culture broth.

Through the catabolic β-oxidation pathway, a fatty acid is iteratively shortened in chain length by two carbons per cycle (Iram & Cronan, 2006). The activation of a free fatty acid for β-oxidation involves its conversion to an acyl-CoA via the acyl-CoA synthetase, FadD. Following this, the first committed step of fatty acid degradation involves the dehydrogenation of the acyl-CoA to an enoyl-CoA by the acyl-CoA dehydrogenase, FadE (Fujita, Matsuoka & Hirooka, 2007; Lennen & Pfleger, 2012). Thus, elimination of *fadE* should prevent exogenously supplied octanoic acid (and endogenously produced fatty acids) from entering the β-oxidation cycle and instead allow the accumulation of octanoyl-CoA via the activity of FadD, effectively eliminating the availability of the competing hexanoyl- and butanoyl-CoA substrates. Plasmid pET21a::*AAT16* was transformed into strain S002 (*E. coli* BL21 (DE3) *ΔfadE*) (Torella et al., 2013) to create strain DLBO1. Expression cultures of DLBO1 were supplemented with 10 mM butanol and 5 mM octanoic acid, and after an 18 h incubation it was found that butyl octanoate was the sole ester product detected in both the culture broth and cell pellet fractions (Fig. 4). The total amount of butyl octanoate produced was 0.75 ± 0.02 mg/L (Fig. 4). Normalising for differences in cell density, this was similar to the amount of butyl octanoate produced by AAT16 in strain C43 (DE3) (Fig. 1), which reached higher cell densities. Thus, although the loss of octanoyl-CoA via β-oxidation had been eliminated—improving the stoichiometry of conversion—to butyl octanoate, there was no improvement in butyl octanoate productivity. This may simply reflect the fact that octanoyl-CoA levels were saturating for enzyme activity in both cases, although it may also be the case that AAT16 was being outcompeted for octanoyl-CoA by other endogenous *E. coli* enzymes. Exogenously supplied fatty acids, upon conversion to their corresponding acyl-CoA, can be directly utilized for phospholipid biosynthesis (Yao & Rock, 2015). Alternatively, this may be the result of slow production of octanoyl-CoA from the free fatty acid. FadD has been shown to have low activity towards fatty acids of fewer than 10 carbons in length (Kameda & Nunn, 1981; Ford & Way, 2015). This hypothesis is supported by the observation of residual free octanoic acid in *E. coli* cultures even 24 h after supplementation (Fig. S11). As *E. coli* lacks the ability to convert exogenously supplied fatty acids to acyl-ACPs for entry into the FAS pathway, the fate of a supplemented fatty acid depends on its conversion to an acyl-CoA via FadD (Cronan & Subrahmanyam, 1998; Yao & Rock, 2015). Further, poor flux of octanoic acid to butyl octanoate presents the opportunity for precursor toxicity via the accumulation of the thioester-CoA, which may affect the rate of product formation. Efficient conversion of octanoic acid to octanoyl-CoA via FadD and of octanoyl-CoA to butyl octanoate via AAT16 is essential to minimize the accumulation of toxic intermediates and/or side reactions.

While β-oxidation perturbation successfully eliminated the production of the undesired shorter chain butyl hexanoate and -butyrate esters from *E. coli* expressing *AAT16* with substrate supplementation, the lack of improvement in total butyl octanoate titre suggests that engineering a system in which butyl octanoate is produced from endogenous octanoyl-CoA will require not only upregulation of octanoic acid production, but also improving its conversion to octanoyl-CoA.

### Engineering endogenous octanoyl-CoA synthesis for butyl octanoate production

**Figure 5:**
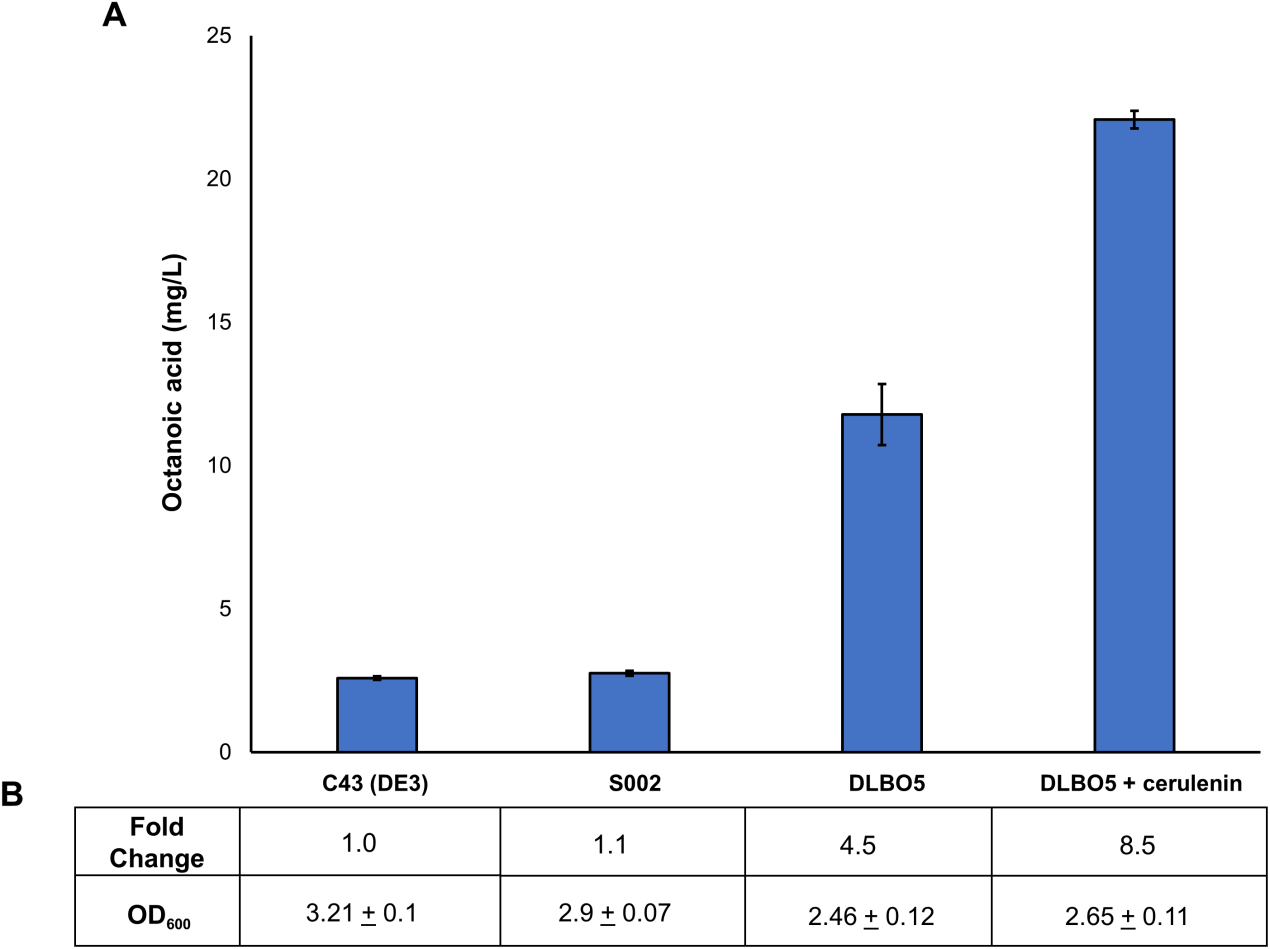
Proportion of free octanoic acid present in the cell pellet fraction of *E. coli* C43 (DE3), S002, DLBO5, and DLBO5 + 4 μg/L cerulenin. (A) Total free octanoic acid detected in the cell pellet from strains C43 (DE3), S002, DLBO5, and DLBO5 + 4 μg/L cerulenin, after 24 h of growth at 30 °C. Fold change is the amount of free octanoic acid relative to the C43 (DE3) control strain (B). Data are the mean ± standard deviation of 3 biological replicates.

**Figure 6:**
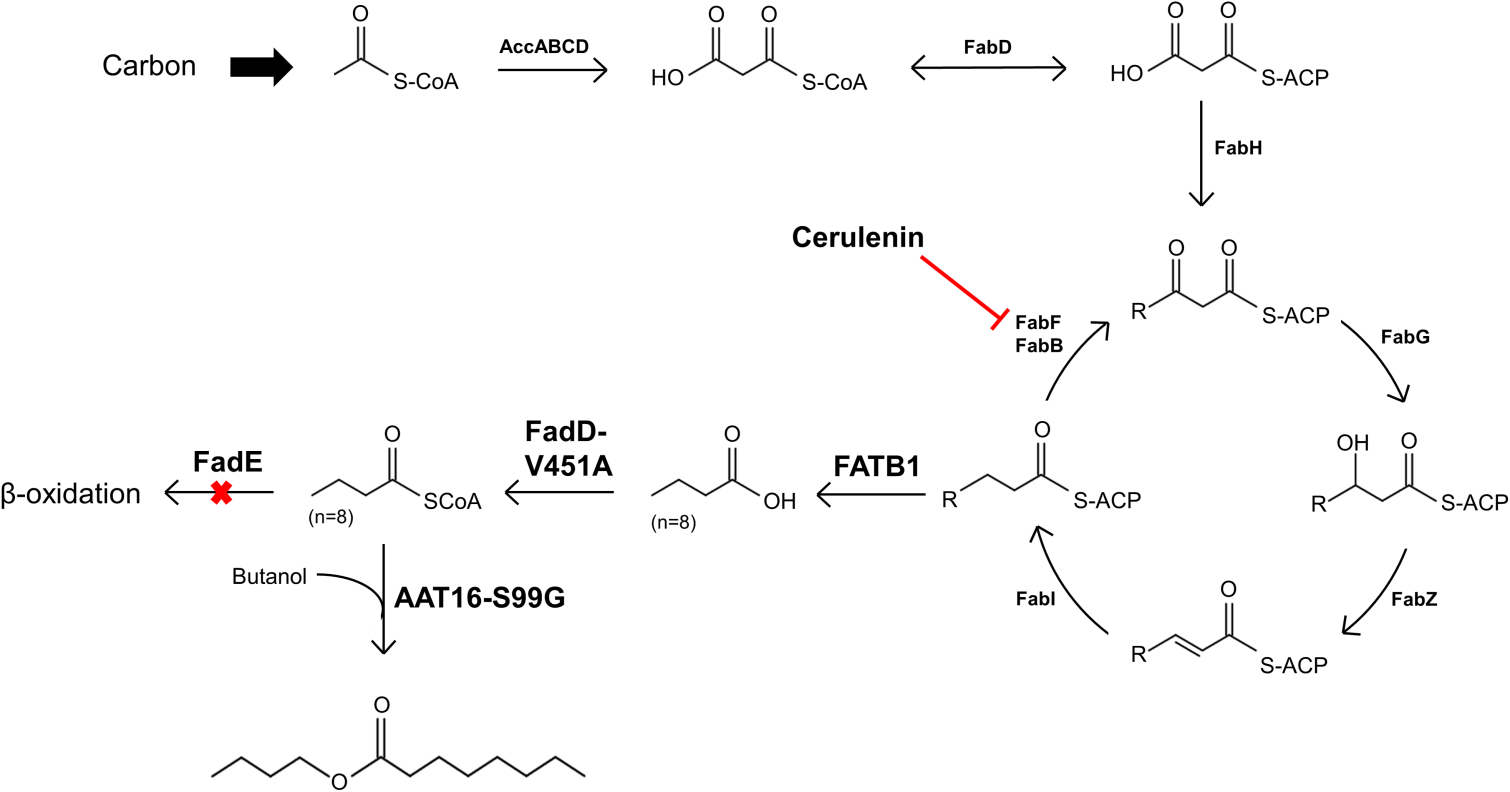
Diagram illustrating the strategy for engineering *E. coli* for the production of butyl octanoate from endogenously produced octanoyl-CoA and exogenously supplied butanol. Diagram of the engineered pathways for the production of butyl octanoate as an end-product from endogenously produced octanoyl-CoA and exogenously supplemented butanol, in *E. coli*. Octanoyl-CoA was produced from de novo fatty acid synthesis via expression of a FATB thioesterase from *C. palustris* and over-expression of an *E. coli* FadD point mutant in a *ΔfadE* background. The antibiotic cerulenin was applied to inhibit fatty acyl-ACP elongation during FAS. Butyl octanoate was produced from endogenous octanoyl-CoA and exogenous butanol via expression of an AAT from *A. chinensis*.

**Figure 7:**
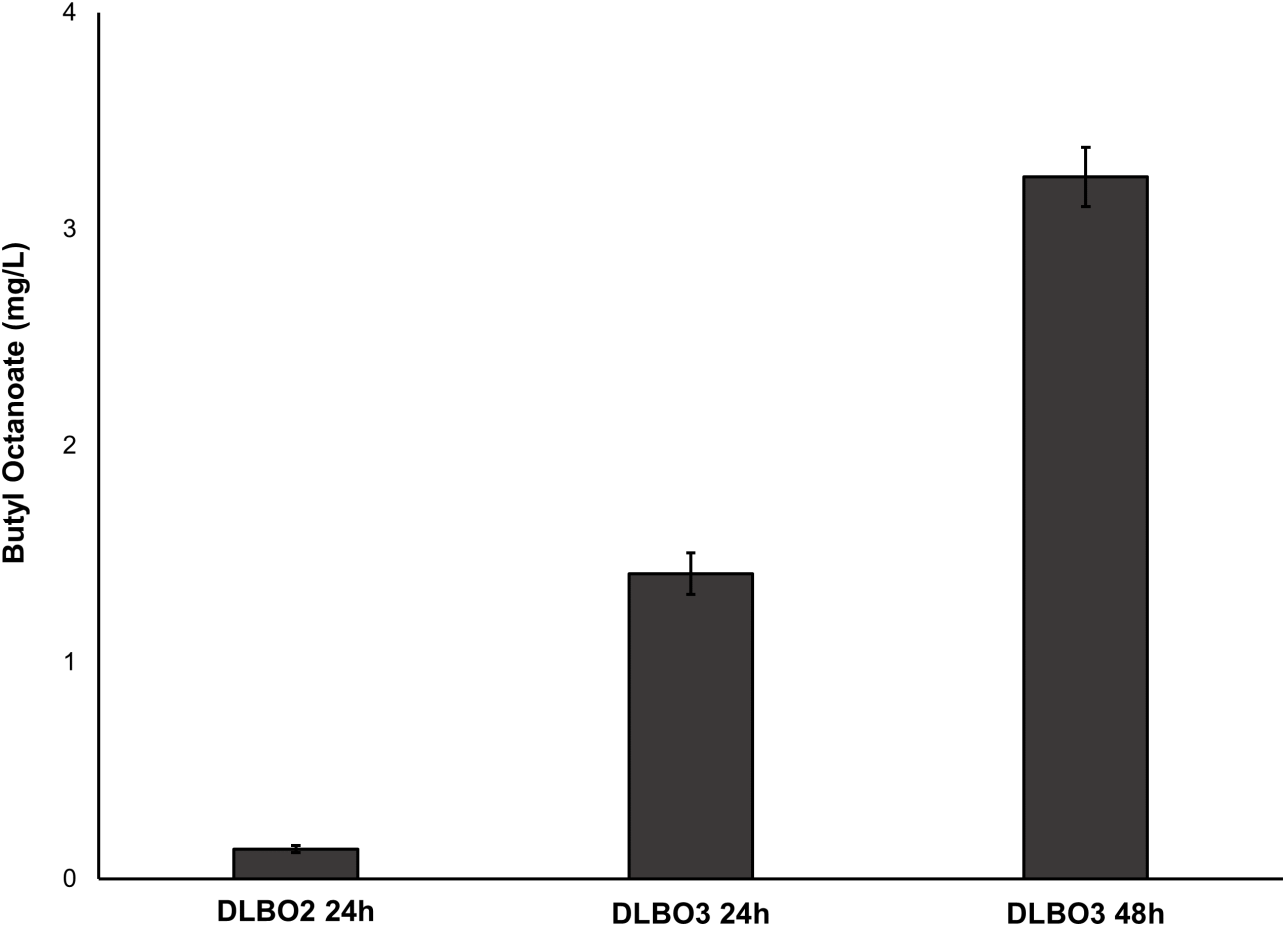
Production of butyl octanoate from strains DLBO2 and DLBO3 after 24 h and 48 h. Butyl octanoate production from strains DLBO2 and DLBO3 after 24 h (DLBO2 and DLBO3) and 48 h (DLBO3). Cultures were supplemented with 10 mM butanol and 4 μg/L cerulenin at time of induction. Values represent total butyl octanoate (cell pellet + culture broth) and are the mean ± standard deviation of 3 biological replicates.

As FAS in *E. coli* typically ends with the production of long-chain acyl-ACPs (C16–C18), engineering fatty acid metabolism to favour the production of medium chain fatty acids (MCFA), such as octanoic acid, requires modification of the FAS pathway. Expression of a thioesterase enzyme (which hydrolyses acyl-ACPs to their corresponding free fatty acids) with activity towards octanoyl-ACP was expected to increase free octanoic acid abundance in the cell. While a number of thioesterases have been characterised as having activity for the C8:0-ACP chain length (Jing et al., 2011; Zheng et al., 2012; Volker et al., 2014; Kirtz et al., 2016; Tan et al., 2018; Feng et al., 2018), the FATB1 thioesterase from *Cuphea palustris* was selected for this work as it demonstrates nearly exclusive substrate specificity for hydrolysing octanoyl-ACP to octanoic acid, and has been used by others for the production of this acid in *E. coli* (Dehesh et al., 1996; Jing et al., 2011; Torella et al., 2013). The sequence of the *FATB1* gene was codon-optimized for expression in *E. coli* and cloned into the pRSETa plasmid to drive expression under the T7 promoter (Table 1). *E. coli* strain S002 (*ΔfadE)* was transformed with plasmid pRSETa::*FATB1* to create strain DLBO5 and this was assessed for free octanoic acid production with and without cerulenin supplementation. Torella et al. (2013) found that the addition of the antibiotic cerulenin—which acts by targeting FabB and FabF to inhibit acyl-ACP elongation during FAS, without affecting the initial condensing enzyme FabH—results in the accumulation of medium chain acyl-ACPs *in vivo* (Jackowski & Rock, 1987; Torella et al., 2013). Cultures of *E. coli* C43 (DE3), S002, DLBO5, and DLBO5 + cerulenin were grown in M9 media + 1% glycerol, induced with IPTG, and incubated for 24 h at 30 °C before analysis. Figure 5 shows the relative abundance of cell associated octanoic acid relative to strain C43 (DE3). From these results, expression of FATB1 in conjunction with cerulenin supplementation resulted in the highest accumulation of intracellular octanoic acid, with 8.5 times more than the control strain. In comparison, strain DLBO5 without cerulenin supplementation gave 4.5 times more octanoic acid, suggesting that cerulenin treatment had a significant impact on medium chain fatty acyl-ACP availability for FATB1 hydrolysis. Converting endogenously produced octanoic acid to the octanoyl-CoA substrate utilised by AAT16 requires the activity of FadD, which we previously suggested might be a potential bottleneck to butyl octanoate production due to the low substrate specificity of this enzyme for MCFA. However, recent work by Ford & Way (2015) has produced an *E. coli* FadD mutant, FadD-V451A, with improved activity towards octanoic acid and decreased activity towards long chain oleic acid. By combining the energetically favourable FATB1-catalyzed hydrolysis of octanoyl-ACP to octanoic acid with its subsequent reactivation to an acyl-CoA—catalysed by the FadD variant with improved activity towards MCFAs—in a β-oxidation perturbed background, fatty acid metabolism may be efficiently directed to octanoyl-CoA synthesis. Figure 6 shows the proposed strategy for butyl octanoate production from endogenous upregulated octanoyl-CoA. To investigate the feasibility of such a strategy plasmid pBEST03 was created (Supplemental Materials; Fig. S6) which included the *E. coli* harmonized coding sequence of AAT16-S99G from *Actinidia chinensis*, and the optimised coding sequences of FATB1 from *Cuphea palustris* and FadD-V451A from *E. coli*, all under the control of T7 promoters to drive high expression. Both plasmids pBEST03 and pET21a::*AAT16-S99G* were co-transformed into strain S002 to create strain DLBO3. *AAT16-S99G* was expressed from both vectors in this strain as it was previously found that this resulted in a small but significant increase in butyl octanoate production (Fig. S9). Cultures of strain DLBO3 and of control strain DLBO2 (S002 + pET21a::*AAT16-S99G*) were induced with IPTG, supplemented with 10 mM butanol and cerulenin, and incubated for 24 or 48 h at 20 °C before analysis. It was found that strain DLBO3 produced 1.5 + 0.1 mg/L of butyl octanoate after 24 h, and 3.3 + 0.1 mg/L after 48 h (Fig. 7). These values represent the total butyl octanoate found in both the culture broth and cell pellet. This is an improvement in production over strains supplemented with exogenous octanoic acid. Multiple factors are potentially contributing to this improvement, including: enhanced conversion of octanoic acid to octanoyl-CoA via the FadD-V451A variant, the potential alleviation of a bottleneck in octanoic acid uptake by the cell through endogenous production of the acid via FATB1, and improved AAT16 abundance and substrate specificity towards octanoyl-CoA. Further work improving/balancing flux through this pathway to resolve potential bottlenecks will be necessary to improve butyl octanoate titres. For instance, Hernández Lozada et al. (2018) recently created a FATB1 variant capable of producing ∼20-fold more octanoic acid in *E. coli* than the FATB1-wt, without cerulenin supplementation. Integration of this FATB1 variant into the DLBO3 system described here would be valuable exercise in determining whether octanoic acid availability is a bottleneck to butyl octanoate production, while concomitantly eliminating the cellular burden imposed by cerulenin supplementation. The control strain, DLBO2, which did not include heterologous expression of FATB1 or FadD-V451A for increased endogenous octanoyl-CoA abundance, did produce a small amount of butyl octanoate. As none was detected from cultures of *E. coli* strain C43 (DE3) harbouring pET21a::*AAT16* supplemented with only butanol (Fig. S8), its production in this strain suggests that the combination of β-oxidation perturbation and cerulenin supplementation are sufficient to supply some octanoyl-CoA for the AAT16-S99G variant to esterify. No ester product was detected at either 24 or 48 h in cultures of strain DLBO2 and DLBO3 where butanol supplementation was excluded (Fig. S13).

Ideally, an *E. coli* strain capable of producing both the butanol and octanoyl-CoA substrates from glucose would be desirable for butyl octanoate production. As such, efforts to produce butyl octanoate from endogenous butanol (with octanoic acid supplementation) via co-expression of *AAT16-S99G* and the modified *Clostridium acetobutylicum* butanol pathway described by Shen et al. (2011) were made in *E. coli.* However, while butanol was produced in excess, only trace amounts of butyl ester were detected (data not shown). Through further investigations it was eventually discovered that transcription of the *AAT16* gene was being inhibited by the expression of the butanol pathway gene, *ter*, from *Treponema denticola*. However, the mechanism by which this was occurring has not yet been determined (Table S1; Fig. S7). As an alternative, butanol production could be attempted through alternative metabolic routes, such as reversed β-oxidation and the keto acid pathway (Dellomonaco et al., 2011; Shen & Liao, 2008).

## Conclusion

This work demonstrates the combined application of protein engineering and fatty acid metabolism engineering for the heterologous production of a single medium chain ester from endogenous acyl-CoA in *E. coli*. Unlike previous studies producing shorter chain esters, an acyl-transferase with the required specificity for octanoyl-CoA was not available at the outset. However, by modelling both the protein structure and amino acid residues which are likely to affect the substrate binding specificity of AAT16 from *Actinidia chinensis*, we have demonstrated that it is possible to improve this enzyme’s specificity for octanoyl-CoA. We have further shown that increasing the pool of octanoyl-CoA by perturbing β oxidation allows the production of butyl octanoate as a single product from cells supplemented with butanol.

Although the low titre of product obtained meant that we recovered it mainly from the cells, the ability to partition across the cell membrane and high relative hydrophobicity means that cells with higher productivities should naturally accumulate this product as a second phase. The production of a pure, non-toxic product which can self-partition offers significant advantages for product recovery. To our knowledge, this represents the first example of AAT mediated synthesis of an ester with acyl chain length >C4 from endogenous acyl-CoA in *E. coli*. This preliminary work represents a first step towards the scalable and cost-effective production of longer medium chain esters from a microbial platform.

## Supplemental Information

10.7717/peerj.6971/supp-1 Supplemental Information 1 Raw Data. AAT activity assays for making BH in pBEST01 pBEST04 and C43 crude cell.

10.7717/peerj.6971/supp-2 Supplemental Information 2 Raw Data. AAT fed butanol and octanoic acid making BB BH and BO.

10.7717/peerj.6971/supp-3 Supplemental Information 3 Raw Data. AAT-SG and pBEST03 in S002 for 24 and 48h.

10.7717/peerj.6971/supp-4 Supplemental Information 4 Raw Data. AAT16 model.

10.7717/peerj.6971/supp-5 Supplemental Information 5 Raw Data. AAT expression in S002 fed butanol and octanoic acid.

10.7717/peerj.6971/supp-6 Supplemental Information 6 Raw Data. MC_AATSGFadE_P trace.

10.7717/peerj.6971/supp-7 Supplemental Information 7 Raw Data. Bradfords and assays of AAT, S99G, and L178F activity for BO esterification.

10.7717/peerj.6971/supp-8 Supplemental Information 8 Raw Data. MC_AATSGFadE_S trace.

10.7717/peerj.6971/supp-9 Supplemental Information 9 Raw Data. AAT fed only butanol making BB and BH.

10.7717/peerj.6971/supp-10 Supplemental Information 10 Raw Data. octanoic acid analysis of FATB1 in C43 and S002 with and without 4ugml Cerulenin.

10.7717/peerj.6971/supp-11 Supplemental Information 11 Raw Data. pBEST04 induced and uninduced for Ter AAT and rrsA qRTPCR.

10.7717/peerj.6971/supp-12 Supplemental Information 12 Raw Data. MC_bbbhbo standards traces.

10.7717/peerj.6971/supp-13 Supplemental Information 13 Raw Data. pBEST01 and pBEST04 induced for AAT and rrsA qRTPCR.

10.7717/peerj.6971/supp-14 Supplemental Information 14 Raw Data. AAT-wt AAT-FI AAT-LF AAT-SG AAT-LF-SG mutants fed butanol and octanoic acid.

10.7717/peerj.6971/supp-15 Supplemental Information 15 Growth assays of *E. coli* in the presence of butyl butyrate, -hexanoate, and -octanoate. Growth assays of *E. coli* C43 (DE3) in the presence of 0, 1, 10, or 100 mM butyl butyrate,–hexanoate, or -octanoate. Cultures were inoculated into LB medium to an initial OD_600_ of ∼0.4 and incubated at 37°C and 250 rpm. OD_600_ was measured every 30 minutes and ester addition occurred 1 hour into the time course. Data are the mean ± standard deviation from three biological replicates.

10.7717/peerj.6971/supp-16 Supplemental Information 16 Coding sequence of the genes used in this study. Full length coding sequences of the genes used in this study. Harmonisation and optimisation was done for the genome of *E. coli*.

10.7717/peerj.6971/supp-17 Supplemental Information 17 Ramachandran plot of AAT16 protein model. Ramachandran plot of AAT16 protein model. The geometric evaluations required to create this plot were performed using MolProbity software (Chen *et al*., 2010; Lovell *et al*., 2003).

10.7717/peerj.6971/supp-18 Supplemental Information 18 Structural superimposition of AAT16-wt with AAT16-S99G, AAT16-L178F, and AAT16-F185I. Structural superimposition of AAT16-wt with AAT16 mutants. **A** superimposition of AAT16-wt (red) and AAT16-S99G (yellow), RMSD of 0.38 Å. **B** superimposition of AAT16-wt (red) and AAT16-L178F (yellow), RMSD of 0.11 Å. **C** superimposition of AAT16-wt (red) and AAT16-F185I (yellow), RMSD of 0.75 Å.

10.7717/peerj.6971/supp-19 Supplemental Information 19 Amino acid sequence alignment of AAT enzymes. Amino acid sequence alignment of fruit or flower derived AAT enzymes from a number of different plant species. Sequences correspond to Genbank accession numbers: Ae (*Actinidia eriantha*) AAT (HO772637); Ban (*Musa sapientum*) AAT (AW025506); Mp (*Malus pumila*) AAT (AY707098); Ac (*Actinidia chinensis*) AAT (HO772640); Cm (*Cucumis melo*) AAT1 (CAA94432), AAT2 (AAL77060), AAT3 (AAW51125), AAT4 (AWW51126); Vp (*Vasconcellea pubescens*) AAT (FJ548611); Cb (*Clarkia breweri*) BEBT (AAN09796), BEAT (AAF04787); Rh (*Rosa hybrid*) AAT (AAW31948); Fa (*Fragaria x ananassa*) SAAT (AAG13130); Fv (*Fragaria vesca*) VAAT (AAN07090). Sequences were aligned using Clustal Omega and BoxShade. Residues of AAT16 that were mutated are highlighted in yellow.

10.7717/peerj.6971/supp-20 Supplemental Information 20 Diagram of steps involved in producing plasmid pBEST03. Diagram illustrating the intermediate steps towards producing plasmid pBEST03 from plasmids pET21a::*AAT16-S99G*, pAG32, pBEST::*GFP*, and a gene fragment containing the *FadD-V451A* gene from *E. coli* and *FATB1* gene from *C. palustris*.

10.7717/peerj.6971/supp-21 Supplemental Information 21 Fold change in expression of the AAT16 gene from plasmids pBEST01 and pBEST04, and fold change in expression of the AAT16 and Ter genes from plasmid pBEST04. Fold change in expression of **A** the *AAT16* gene in *E. coli* expressing either plasmid pBEST01 (p15A, HygB^R^, T7, *AAT16*_Ac_) or pBEST04 (p15A, HygB^R^, T7, *AAT16*_Ac_, T7, *Ter_Td_*, *Fdh*_Cb_), **B** fold change in expression of either the *AAT16* or the *Ter* gene in *E. coli* expressing plasmid pBEST04. Values are given as fold change in expression relative to wildtype signal–the no template control (=1.0)–using 16S ribosomal RNA (rrsA) as a reference gene. Data is the mean + standard deviation of three biological replicates.

10.7717/peerj.6971/supp-22 Supplemental Information 22 Production of butyl butyrate and -hexanoate from *E. coli* C43 (DE3) expressing AAT16 upon exogenous supplementation of 10 mM butanol. Total production of butyl butyrate and butyl hexanoate from *E. coli* C43 (DE3) expressing the harmonised AAT16 from *A. chinensis* upon exogenous addition of only 10 mM butanol. Products were analysed from both the culture supernatant and cell pellet after 18h of growth at 20°C. Data are the mean ± standard deviation from three biological replicates.

10.7717/peerj.6971/supp-23 Supplemental Information 23 Production of butyl octanoate from* E. coli* harbouring either pBEST02 or pBEST02 + pET21a::AAT16-S99G upon exogenous addition of 10 mM butanol and 5 mM octanoic acid. Production of butyl octanoate from *E. coli* C43 (DE3) harbouring either pBEST02 or pBEST02 + pET21a::*AAT16-S99G* upon exogenous addition of 10 mM butanol and 5 mM octanoic acid. Products were analysed from both the culture supernatant and cell pellet after 18h of growth at 20°C. Data are the mean ± standard deviation from three biological replicates.

10.7717/peerj.6971/supp-24 Supplemental Information 24 SDS-PAGE gel demonstrating the expression of AAT-wt, AAT-F185I, AAT-S99G, and AAT-L178F in *E. coli* C43 (DE3). SDS-PAGE gel demonstrating the expression of AAT-wt, AAT-F185I, AAT-S99G, and AAT-L178F after 18 hours with (+) or without (-) IPTG induction.

10.7717/peerj.6971/supp-25 Supplemental Information 25 Gas chromatogram showing residual octanoic acid present at time of product analysis of strain DLBO1. Gas chromatogram showing residual octanoic acid at time of product analysis of strain DLBO1 supplemented with 10 mM butanol and 5 mM octanoic acid. Cultures were incubated for 18h at 20°C.

10.7717/peerj.6971/supp-26 Supplemental Information 26 Spatial distribution of substrates butanol and octanoyl-CoA in the active site of AAT16-S99G-L178F. Spatial distribution of substrates butanol and octanoyl-CoA in the active site of AAT16-S99G-L178F. Distance (Å) from the ɛ nitrogen of H167 to the carbonyl group of the octanoyl-CoA and the hydroxyl group of the butanol is indicated.

10.7717/peerj.6971/supp-27 Supplemental Information 27 Gas chromatograms showing lack of butyl ester production from strain DLBO2 and DLBO3 supplemented with only 5 mM octanoic acid after 24h of growth at 20°C. Gas chromatograms showing lack of butyl ester production from strain DLBO2 **B** and DLBO3 **C** supplemented with only 5 mM octanoic acid after 24h of growth at 20°C. Panel **A** shows the retention times of butyl butyrate, -hexanoate, and -octanoate standards.

10.7717/peerj.6971/supp-28 Supplemental Information 28 *In vitro* activity of Ter and AAT16 in crude cell extracts of wild type *E. coli* C43 (DE3), and *E. coli* C43 (DE3) harbouring either pBEST01 or pBEST04. Activity is the mean + standard deviation of four replicates. Ter assay performed at 30°C and 340 nm using 200 μmol NADH, 2 μmol FAD, and 200 μmol crotonyl-CoA. AAT16 assay performed at 30°C for 30 minutes using 10 mM butanol and 0.75 mM hexanoyl-CoA.

10.7717/peerj.6971/supp-29 Supplemental Information 29 Description of *E. coli* plasmids used in the supplementary work of this study. In the plasmid description, subscript letters refer to the source of the gene as follows: Ac = *Actinidia chinensis*, Td = *Treponema denticola*, Cb = *Candida boidinii*.

10.7717/peerj.6971/supp-30 Supplemental Information 30 Oligonucleotide primers used in this study. All primers were synthesised and purchased from Eurofins Genomics (Germany). Oligonucleotide primers used in this study.

10.7717/peerj.6971/supp-31 Supplemental Information 31 Supplementary Methods.

## Additional Information and Declarations

### Data Availability

The following information was supplied regarding data availability: All the raw data is available in the Supplemental Files.

## References

[ref-1] Abagyan R, Totrov M, Kuznetsov D (1994). ICM—A new method for protein modeling and design: applications to docking and structure prediction from the distorted native conformation. Journal of Computational Chemistry.

[ref-2] Aharoni A, Keizer LCP, Bouwmeester HJ, Sun Z, Alvarez-Huerta M, Verhoeven HA, Blaas J, Van Houwelingen AMML, De Vos RCH, Van Der Voet H, Jansen RC, Guis M, Mol J, Davis RW, Schena M, Van Tunen AJ, O’connell AP (2000). Identification of the SAAT gene involved in strawberry flavor biogenesis by use of DNA microarrays. Plant Cell.

[ref-3] Arnold K, Bordoli L, Kopp J, Schwede T (2006). The SWISS-MODEL workspace: a web-based environment for protein structure homology modelling. Bioinformatics.

[ref-4] Atsumi S, Hanai T, Liao JC (2008). Non-fermentative pathways for synthesis of branched-chain higher alcohols as biofuels. Nature.

[ref-5] Beekwilder J, Alvarez-Huerta M, Neef E, Verstappen FW, Bouwmeester HJ, Aharoni A (2004). Functional characterization of enzymes forming volatile esters from strawberry and banana. Plant Physiology.

[ref-6] Bradford M (1976). A rapid and sensitive method for the quantitation of microgram quantities of protein utilizing the principle of protein-dye binding. Analytical Biochemistry.

[ref-7] Chen VB, Arendall WB, Headd JJ, Keedy DA, Immormino RM, Kapral GJ, Murray LW, Richardson JS, Richardson DC (2010). MolProbity: all-atom structure validation for macromolecular crystallography. Acta Crystallographica Section D Biological Crystallography.

[ref-8] Clomburg JM, Blankschien MD, Vick JE, Chou A, Kim S, Gonzalez R (2015). Integrated engineering of β-oxidation reversal and ω-oxidation pathways for the synthesis of medium chain ω-functionalized carboxylic acids. Metabolic Engineering.

[ref-9] Costello PJ, Siebert TE, Solomon MR, Bartowsky EJ (2013). Synthesis of fruity ethyl esters by acyl coenzyme A: alcohol acyltransferase and reverse esterase activities in *Oenococcus oeni* and *Lactobacillus plantarum*. Journal of Applied Microbiology.

[ref-10] Cronan JE, Subrahmanyam S (1998). FadR, transcriptional co-ordination of metabolic expediency. Molecular Microbiology.

[ref-11] D’Auria JC, Chen F, Pichersky E (2002). Characterization of an acyltransferase capable of synthesizing benzylbenzoate and other volatile esters in flowers and damaged leaves of *Clarkia breweri*. Plant Physiology.

[ref-89] Dellomonaco C, Clomburg JM, Miller EN, Gonzalez R (2011). Engineered reversal of the beta-oxidation cycle for the synthesis of fuels and chemicals. Nature.

[ref-12] De Jong BW, Shi S, Siewers V, Nielsen J (2014). Improved production of fatty acid ethyl esters in *Saccharomyces cerevisiae* through up-regulation of the ethanol degradation pathway and expression of the heterologous phosphoketolase pathway. Microbial Cell Factories.

[ref-13] De Souza MCM, Dos Santos KP, Freire RM, Barreto ACH, Fechine PBA, Gonçalves LRB (2017). Production of flavor esters catalyzed by Lipase B from *Candida antarctica* immobilized on magnetic nanoparticles. Brazilian Journal of Chemical Engineering.

[ref-14] Dehesh K, Edwards P, Hayes T, Cranmer AM, Fillatti J (1996). Two novel thioesterases are key determinants of the bimodal distribution of acyl chain length of *Cuphea palustris* seed oil. Plant Physiology.

[ref-15] Demain AL (2000). Small bugs, big business: the economic power of the microbe. Biotechnology Advances.

[ref-16] Demain AL (2006). From natural products discovery to commercialization: a success story. Journal of Industrial Microbiology and Biotechnology.

[ref-17] Dhake KP, Thakare DD, Bhanage BM (2013). Lipase: a potential biocatalyst for the synthesis of valuable flavour and fragrance ester compounds. Flavour and Fragrance Journal.

[ref-18] Duan Y, Zhu Z, Cai K, Tan X, Lu X (2011). De novo biosynthesis of biodiesel by *Escherichia coli* in optimized fed-batch cultivation. PLOS ONE.

[ref-19] Dudareva N, Pichersky E, Gershenzon J (2004). Biochemistry of plant volatiles. Plant Physiology.

[ref-20] Elbahloult Y, Steinbüchel A (2010). Pilot-scale production of fatty acid ethyl esters by an engineered *Escherichia coli strain* harboring the p(Microdiesel) plasmid. Applied and Environmental Microbiology.

[ref-21] El-Sharkawy I, Manríquez D, Flores FB, Regad F, Bouzayen M, Latché A, Pech J-C (2005). Functional characterization of a melon alcohol acyl-transferase gene family involved in the biosynthesis of ester volatiles. Identification of the crucial role of a threonine residue for enzyme activity. Plant Molecular Biology.

[ref-22] Feng Y, Zhang Y, Wang Y, Liu J, Liu Y, Cao X, Xui S (2018). Tuning of acyl-ACP thioesterase activity directed for tailored fatty acid synthesis. Applied Microbiology and Biotechnology.

[ref-95] Ford TJ, Way JC (2015). Enhancement of *E. coli* acyl-CoA synthetase FadD activity on medium chain fatty acids. PeerJ.

[ref-93] Fujita Y, Matsuoka H, Hirooka K (2007). Regulation of fatty acid metabolism in bacteria. Molecular Microbiology.

[ref-23] Galaz S, Morales-Quintana L, Moya-León MA, Herrera R (2013). Structural analysis of the alcohol acyltransferase protein family from *Cucumis melo* shows that enzyme activity depends on an essential solvent channel. FEBS Journal.

[ref-91] Goldstein AL, McCusker JH (1999). Three new dominant drug resistance cassettes for gene disruption in Saccharomyces cerevisiae. Yeast.

[ref-24] Goulet C, Kamiyoshihara Y, Lam NB, Richard T, Taylor MG, Tieman DM, Klee HJ (2015). Divergence in the enzymatic activities of a tomato and *Solanum pennellii* alcohol acyltransferase impacts fruit volatile ester composition. Molecular Plant.

[ref-88] Grand View Research (2017). Flavors and fragrances market analysis by product (Natural, Aroma), by application (Flavors, Fragrances), by region (North America, Europe, APAC, MEA, Central & South America), & Segment Forecasts. https://www.grandviewresearch.com/industry-analysis/flavors-fragrances-market/toc.

[ref-25] Günther CS, Chervin C, Marsh KB, Newcomb RD, Souleyre EJF (2011). Characterisation of two alcohol acyltransferases from kiwifruit (*Actinidia spp*.) reveals distinct substrate preferences. Phytochemistry.

[ref-26] Guo D, Pan H, Li X (2015). Metabolic engineering of *Escherichia coli* for production of biodiesel from fatty alcohols and acetyl-CoA. Applied Microbiology and Biotechnology.

[ref-27] Harada M, Ueda Y, Iwata T (1985). Purification and some properties of alcohol acetyltransferase from banana fruit. Plant and Cell Physiology.

[ref-28] Heckman KL, Pease LR (2007). Gene splicing and mutagenesis by PCR-driven overlap extension. Nature Protocols.

[ref-29] Hernández I, Molenaar D, Beekwilder J, Bouwmeester H, Van Hylckama Vlieg JET (2007). Expression of plant flavor genes in *Lactococcus lactis*. Applied and Environmental Microbiology.

[ref-30] Hernández Lozada NJ, Lai R-Y, Simmons TR, Thomas KA, Chowdhury R, Maranas CD, Pfleger BF (2018). Highly active C_8_-acyl-ACP thioesterase variant isolated by a synthetic selection strategy. ACS Synthetic Biology.

[ref-31] Horton CE, Huang KX, Bennett GN, Rudolph FB (2003). Heterologous expression of the *Saccharomyces cerevisiae* alcohol acetyltransferase genes in *Clostridium acetobutylicum* and *Escherichia coli* for the production of isoamyl acetate. Journal of Industrial Microbiology and Biotechnology.

[ref-32] Inui M, Suda M, Kimura S, Yasuda K, Suzuki H, Toda H, Yamamoto S, Okino S, Suzuki N, Yukawa H (2008). Expression of *Clostridium acetobutylicum* butanol synthetic genes in *Escherichia coli*. Applied Microbiology and Biotechnology.

[ref-33] Iram SH, Cronan JE (2006). The β-oxidation systems of *Escherichia coli* and *Salmonella enterica* are not functionally equivalent. Journal of Bacteriology.

[ref-92] Janßen HJ, Steinbüchel A (2014). Fatty acid synthesis in Escherichia coli and its applications towards the production of fatty acid based biofuels. Biotechnology for Biofuels.

[ref-34] Jackowski S, Rock CO (1987). Acetoacetyl-acyl carrier protein synthase, a potential regulator of fatty acid biosynthesis in bacteria. Journal of Biological Chemistry.

[ref-35] Jawed K, Mattam AJ, Fatma Z, Wajid S, Abdin MZ, Yazdani SS (2016). Engineered production of short chain fatty acid in *Escherichia coli* using fatty acid synthesis pathway. PLOS ONE.

[ref-36] Jin Z, Ntwali J, Han S-Y, Zheng S-P, Lin Y (2012). Production of flavor esters catalyzed by CALB-displaying *Pichia pastoris* whole-cells in a batch reactor. Journal of Biotechnology.

[ref-37] Jing F, Cantu DC, Tvaruzkova J, Chipman JP, Nikolau BJ, Yandeau-Nelson MD, Reilly PJ (2011). Phylogenetic and experimental characterization of an acyl-ACP thioesterase family reveals significant diversity in enzymatic specificity and activity. BMC Biochemistry.

[ref-38] Kalscheuer R, Stölting T, Steinbüchel A (2006b). Microdiesel: *Escherichia coli* engineered for fuel production. Microbiology.

[ref-39] Kalscheuer R, Stöveken T, Luftmann H, Malkus U, Reichelt R, Steinbüchel A (2006a). Neutral lipid biosynthesis in engineered *Escherichia coli*: Jojoba oil-like wax esters and fatty acid butyl esters. Applied and Environmental Microbiology.

[ref-94] Kameda K, Nunn W (1981). Purification and characterization of acyl coenzyme A synthetase from Escherichia coli. Journal of Biological Chemistry.

[ref-40] Kirtz M, Klebensberger J, Otte KB, Richter SM, Hauer B (2016). Production of ω-hydroxy octanoic acid with *Escherichia coli*. Journal of Biotechnology.

[ref-42] Lallemand LA, Zubieta C, Lee SG, Wang Y, Acajjaoui S, Timmins J, McSweeney S, Jez JM, McCarthy JG, McCarthy AA (2012). A structural basis for the biosynthesis of the major chlorogenic acids found in coffee. Plant Physiology.

[ref-43] Layton DS, Trinh CT (2014). Engineering modular ester fermentative pathways in *Escherichia coli*. Metabolic Engineering.

[ref-44] Layton DS, Trinh CT (2016). Expanding the modular ester fermentative pathways for combinatorial biosynthesis of esters from volatile organic acids. Biotechnology and Bioengineering.

[ref-45] Lennen RM, Pfleger BF (2012). Engineering *Escherichia coli* to synthesize free fatty acids. Trends in Biotechnology.

[ref-46] Leslie AGW (1990). Refined crystal structure of type III chloramphenicol acetyltransferase at 1·75 Å resolution. Journal of Molecular Biology.

[ref-47] Lewendon A, Murray IA, Shaw WV, Gibbs MR, Leslie GW (1994). Replacement of catalytic histidine-195 of chloramphenicol acetyltransferase: evidence for a general base role for glutamate. Biochemistry.

[ref-48] Lucchetta L, Manriquez D, El-Sharkawy I, Flores FB, Sanchez-Bel P, Zouine M, Ginies C, Bouzayen M, Rombaldi C, Pech JC, Latché A (2007). Biochemical and catalytic properties of three recombinant alcohol acyltransferases of melon. Sulfur-containing ester formation, regulatory role of CoA-SH in activity, and sequence elements conferring substrate preference. Journal of Agricultural and Food Chemistry.

[ref-49] Lüthy R, Bowie JU, Eisenberg D (1992). Assessment of protein models with three-dimensional profiles. Nature.

[ref-50] Ma X, Koepke J, Panjikar S, Fritzsch G, Stöckigt J (2005). Crystal structure of vinorine synthase, the first representative of the BAHD superfamily. Journal of Biological Chemistry.

[ref-51] Menendez-Bravo S, Comba S, Gramajo H, Arabolaza A (2017). Metabolic engineering of microorganisms for the production of structurally diverse esters. Applied Microbiology and Biotechnology.

[ref-52] Morales-Quintana L, Fuentes L, Gaete-Eastman C, Herrera R, Moya-León MA (2011). Structural characterization and substrate specificity of VpAAT1 protein related to ester biosynthesis in mountain papaya fruit. Journal of Molecular Graphics and Modelling.

[ref-53] Morales-Quintana L, Nuñez-Tobar MX, Moya-León MA, Herrera R (2013). Molecular dynamics simulation and site-directed mutagenesis of alcohol acyltransferase: a proposed mechanism of catalysis. Journal of Chemical Information and Modeling.

[ref-54] Navarro-Retamal C, Gaete-Eastman C, Herrera R, Caballero J, Alzate-Morales JH (2016). Structural and affinity determinants in the interaction between alcohol acyltransferase from *F. x ananassa* and several alcohol substrates: a computational study. PLOS ONE.

[ref-55] Olias JM, Sanz C, Rios JJ, Perez AG, Rouseff RL, Leahy MM (1995). Substrate specificity of alcohol acyltransferase from strawberry and banana fruits. Fruit Flavors.

[ref-56] Onuki S, Koziel JA, van Leeuwen J, Jenks WS, Grewell DA, Cai L (2008). Ethanol production, purification, and analysis techniques: a review. https://lib.dr.iastate.edu/abe_eng_conf/68.

[ref-57] Orlova I, Marshall-Colon A, Schnepp J, Wood B, Varbanova M, Fridman E, Blakeslee JJ, Peer WA, Murphy AS, Rhodes D, Pichersky E, Dudareva N (2006). Reduction of Benzenoid synthesis in petunia flowers reveals multiple pathways to benzoic acid and enhancement in auxin transport. Plant Cell Online.

[ref-58] Park YC, Shaffer CEH, Bennett GN (2009). Microbial formation of esters. Applied Microbiology and Biotechnology.

[ref-60] Rodriguez GM, Tashiro Y, Atsumi S (2014). Expanding ester biosynthesis in *Escherichia coli*. Nature Chemical Biology.

[ref-61] Röttig A, Zurek PJ, Steinbüchel A (2015). Assessment of bacterial acyltransferases for an efficient lipid production in metabolically engineered strains of *E. coli*. Metabolic Engineering.

[ref-62] Schrader J, Etschmann MMW, Sell D, Hilmer JM, Rabenhorst J (2004). Applied biocatalysis for the synthesis of natural flavour compounds—current industrial processes and future prospects. Biotechnology Letters.

[ref-96] Shen CR, Lan EI, Dekishima Y, Baez A, Cho KM, Liao JC (2011). Driving forces enable high-titer anaerobic 1-butanol synthesis in Escherichia coli. Applied and Environmental Microbiology.

[ref-63] Shen CR, Liao JC (2008). Metabolic engineering of *Escherichia coli* for 1-butanol and 1-propanol production via the keto-acid pathways. Metabolic Engineering.

[ref-64] Shi S, Valle-Rodríguez JO, Khoomrung S, Siewers V, Nielsen J (2012). Functional expression and characterization of five wax ester synthases in *Saccharomyces cerevisiae* and their utility for biodiesel production. Biotechnology for Biofuels.

[ref-90] Shin J, Noireaux V (2010). Efficient cell-free expression with the endogenous *E. coli* RNA polymerase and sigma factor 70. Journal of Biological Engineering.

[ref-65] Sose MT, Bansode SR, Rathod VK (2017). Solvent free lipase catalyzed synthesis of butyl caprylate. Journal of Chemical Sciences.

[ref-66] Souleyre EJF, Greenwood DR, Friel EN, Karunairetnam S, Newcomb RD (2005). An alcohol acyl transferase from apple (cv. Royal Gala), MpAAT1, produces esters involved in apple fruit flavor. FEBS Journal.

[ref-67] Steen EJ, Kang Y, Bokinsky G, Hu Z, Schirmer A, McClure A, Del Cardayre SB, Keasling JD (2010). Microbial production of fatty-acid-derived fuels and chemicals from plant biomass. Nature.

[ref-68] Stöveken T, Kalscheuer R, Malkus U, Reichelt R, Steinbüchel A (2005). The wax ester synthase/acyl coenzyme A:diacylglycerol acyltransferase from *Acinetobacter sp*. strain ADP1: characterization of a novel type of acyltransferase. Journal of Bacteriology.

[ref-69] Suzuki H, Nakayama T, Nishino T (2003). Proposed mechanism and functional amino acid residues of malonyl-CoA:anthocyanin 5-o-glucoside-6‴-O-malonyltransferase from flowers of *Salvia splendens*, a member of the versatile plant acyltransferase family. Biochemistry.

[ref-70] Tai YS, Xiong M, Zhang K (2015). Engineered biosynthesis of medium-chain esters in *Escherichia coli*. Metabolic Engineering.

[ref-71] Tan Z, Yoon JM, Chowdhury A, Burdick K, Jarboe LR, Maranas CD, Shanks JV (2018). Engineering of *E. coli* inherent fatty acid biosynthesis capacity to increase octanoic acid production. Biotechnology for Biofuels.

[ref-72] Tanner KG, Trievel RC, Kuo MH, Howard RM, Berger SL, Allis CD, Marmorstein R, Denut JM (1999). Catalytic mechanism and function of invariant glutamic acid 173 from the histone acetyltransferase GCN5 transcriptional coactivator. Journal of Biological Chemistry.

[ref-73] Torella JP, Ford TJ, Kim SN, Chen AM, Way JC, Silver PA (2013). Tailored fatty acid synthesis via dynamic control of fatty acid elongation. Proceedings of the National Academy of Sciences of the United States of America.

[ref-74] Trinh CT, Unrean P, Srienc F (2008). Minimal *Escherichia coli* cell for the most efficient production of ethanol from hexoses and pentoses. Applied and Environmental Microbiology.

[ref-76] Vadali RV, Horton CE, Rudolph FB, Bennett GN, San KY (2004). Production of isoamyl acetate in ackA-pta and/or ldh mutants of *Escherichia coli* with overexpression of yeast ATF2. Applied Microbiology and Biotechnology.

[ref-77] Verstrepen KJ, Van Laere SDM, Vanderhaegen BMP, Derdelinckx G, Dufour J, Pretorius IS, Winderickx J, Thevelein JM, Delvaux FR (2003). Expression levels of the yeast alcohol acetyltransferase genes ATF1, Lg-ATF1, and ATF2 control the formation of a broad range of volatile esters. Society.

[ref-78] Volker AR, Gogerty DS, Bartholomay C, Hennen-Bierwagen T, Zhu H, Bobik TA (2014). Fermentative production of short-chain fatty acids in *Escherichia coli*. Microbiology.

[ref-79] Walker AM, Hayes RP, Youn B, Vermerris W, Sattler SE, Kang C (2013). Elucidation of the structure and reaction mechanism of sorghum hydroxycinnamoyltransferase and its structural relationship to other coenzyme a-dependent transferases and synthases. Plant Physiology.

[ref-80] Welsh FW, Murray WD, Williams RE, Katz I (1989). Microbiological and enzymatic production of flavor and fragrance chemicals. Critical Reviews in Biotechnology.

[ref-81] Wu J, Zhang X, Xia X, Dong M (2017). A systematic optimization of medium chain fatty acid biosynthesis via the reverse beta-oxidation cycle in *Escherichia coli*. Metabolic Engineering.

[ref-82] Xia X (2013). DAMBE5: a comprehensive software package for data analysis in molecular biology and evolution. Molecular Biology and Evolution.

[ref-83] Yamauchi H, Hasuo T, Amachi T, Akita O, Hara S, Yoshizawals K (1989). Purification and characterization of Acyl Coenzyme A: alcohol Acyltransferase of *Neurospora sp*. Agricultural and Biological Chemistry.

[ref-84] Yao J, Rock CO (2015). How bacterial pathogens eat host lipids: implications for the development of fatty acid synthesis therapeutics. Journal of Biological Chemistry.

[ref-85] Zhang F, Carothers JM, Keasling JD (2012). Design of a dynamic sensor-regulator system for production of chemicals and fuels derived from fatty acids. Nature Biotechnology.

[ref-86] Zhang H, Wang P, Qi Q (2006). Molecular effect of FadD on the regulation and metabolism of fatty acid in *Escherichia coli*. FEMS Microbiology Letters.

[ref-87] Zheng Y, Li L, Liu Q, Qin W, Yang J, Cao Y, Jiang X, Zhao G, Xian M (2012). Boosting the free fatty acid synthesis of *Escherichia coli* by expression of a cytosolic *Acinetobacter baylyi* thioesterase. Biotechnology for Biofuels.

