# Supplementary figures and images for: Engineering Escherichia coli for the production of butyl octanoate from endogenous octanoyl-CoA
Source: PeerJ. 2019 Jul 1;7:e6971. doi: 10.7717/peerj.6971 (PMC6610577; doi:10.7717/peerj.6971)

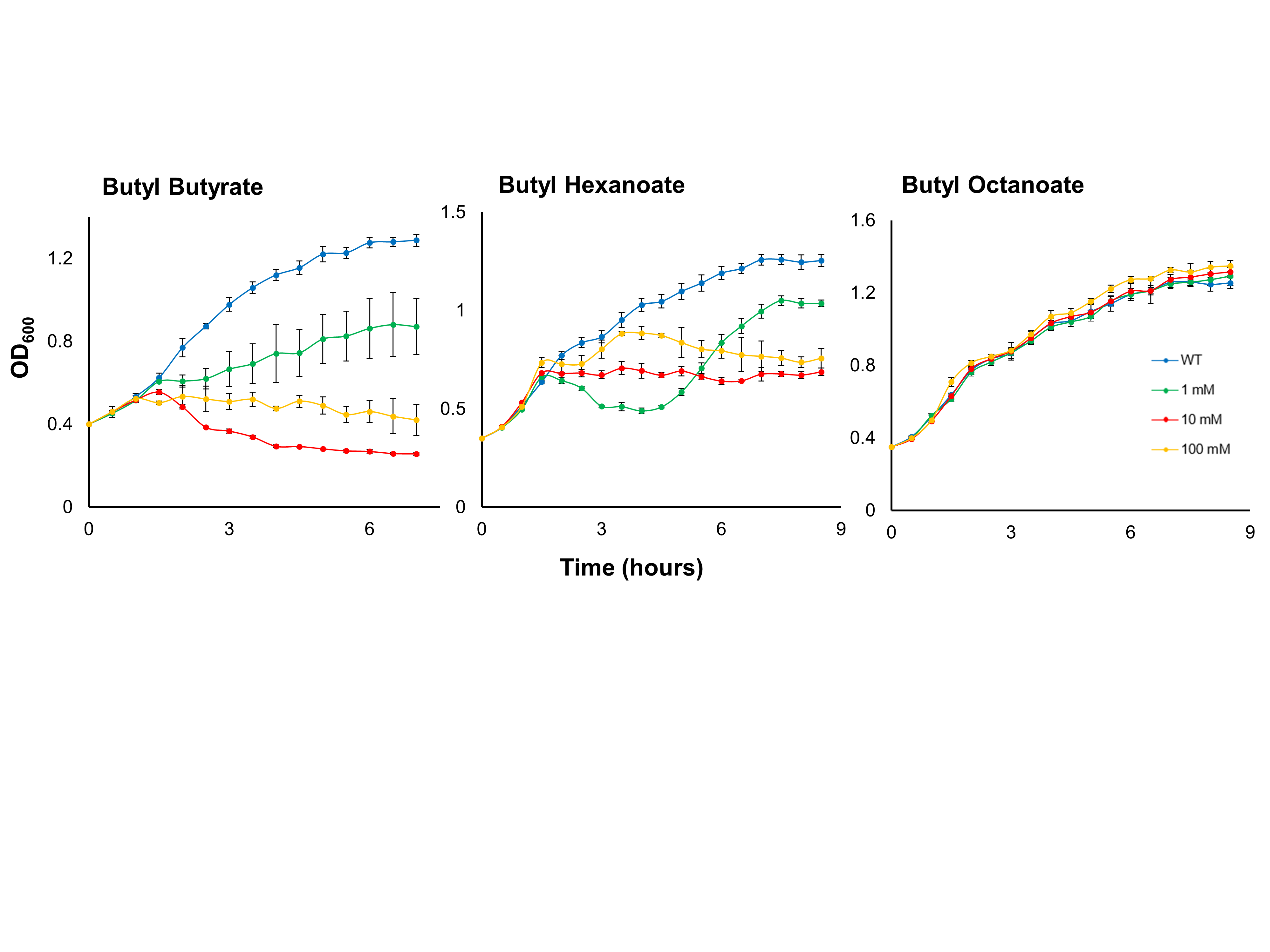

Supplement: Supplemental Information 15 — Growth assays of E. coli C43 (DE3) in the presence of 0, 1, 10, or 100 mM butyl butyrate,–hexanoate, or -octanoate. Cultures were inoculated into LB medium to an initial OD600 of ∼0.4 and incubated at 37°C and 250 rpm. OD600 was measured every 30 minutes and ester addition occurred 1 hour into the time course. Data are the mean ± standard deviation from three biological replicates. [file peerj-07-6971-s015.png]

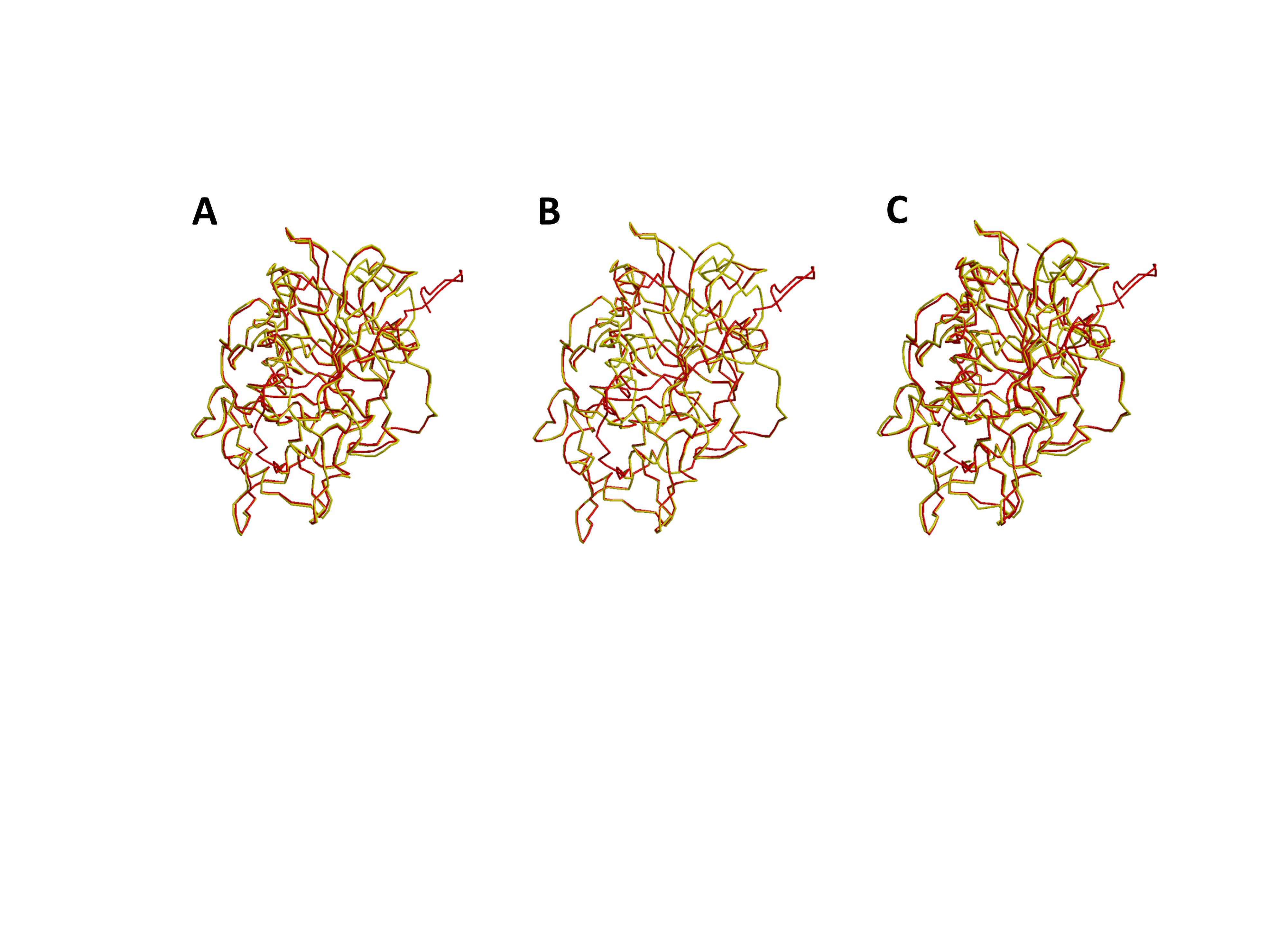

Supplement: Supplemental Information 18 — Structural superimposition of AAT16-wt with AAT16 mutants. A superimposition of AAT16-wt (red) and AAT16-S99G (yellow), RMSD of 0.38 Å. B superimposition of AAT16-wt (red) and AAT16-L178F (yellow), RMSD of 0.11 Å. C superimposition of AAT16-wt (red) and AAT16-F185I (yellow), RMSD of 0.75 Å. [file peerj-07-6971-s018.png]

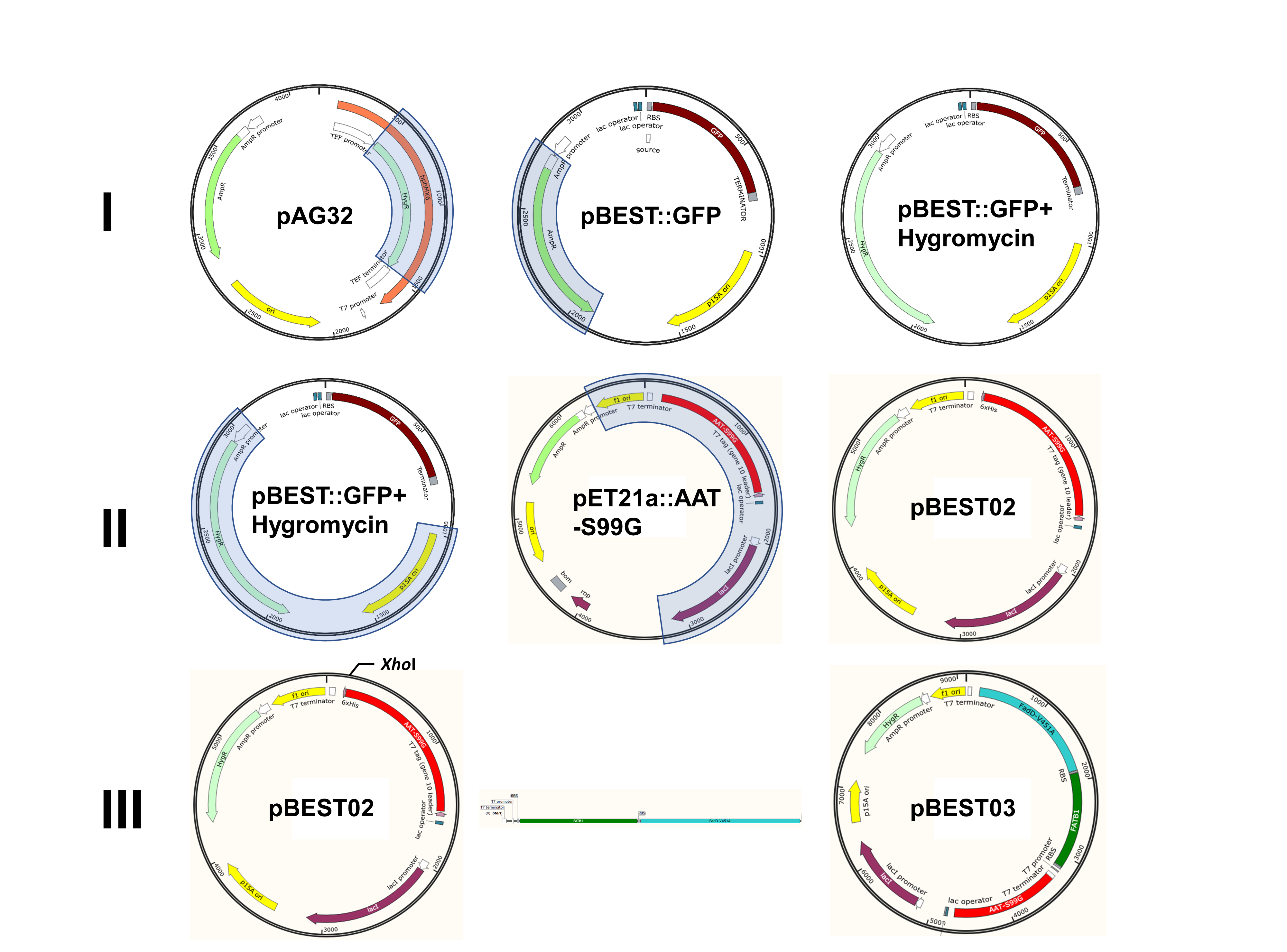

Supplement: Supplemental Information 20 — Diagram illustrating the intermediate steps towards producing plasmid pBEST03 from plasmids pET21a::AAT16-S99G, pAG32, pBEST::GFP, and a gene fragment containing the FadD-V451A gene from E. coli and FATB1 gene from C. palustris. [file peerj-07-6971-s020.png]

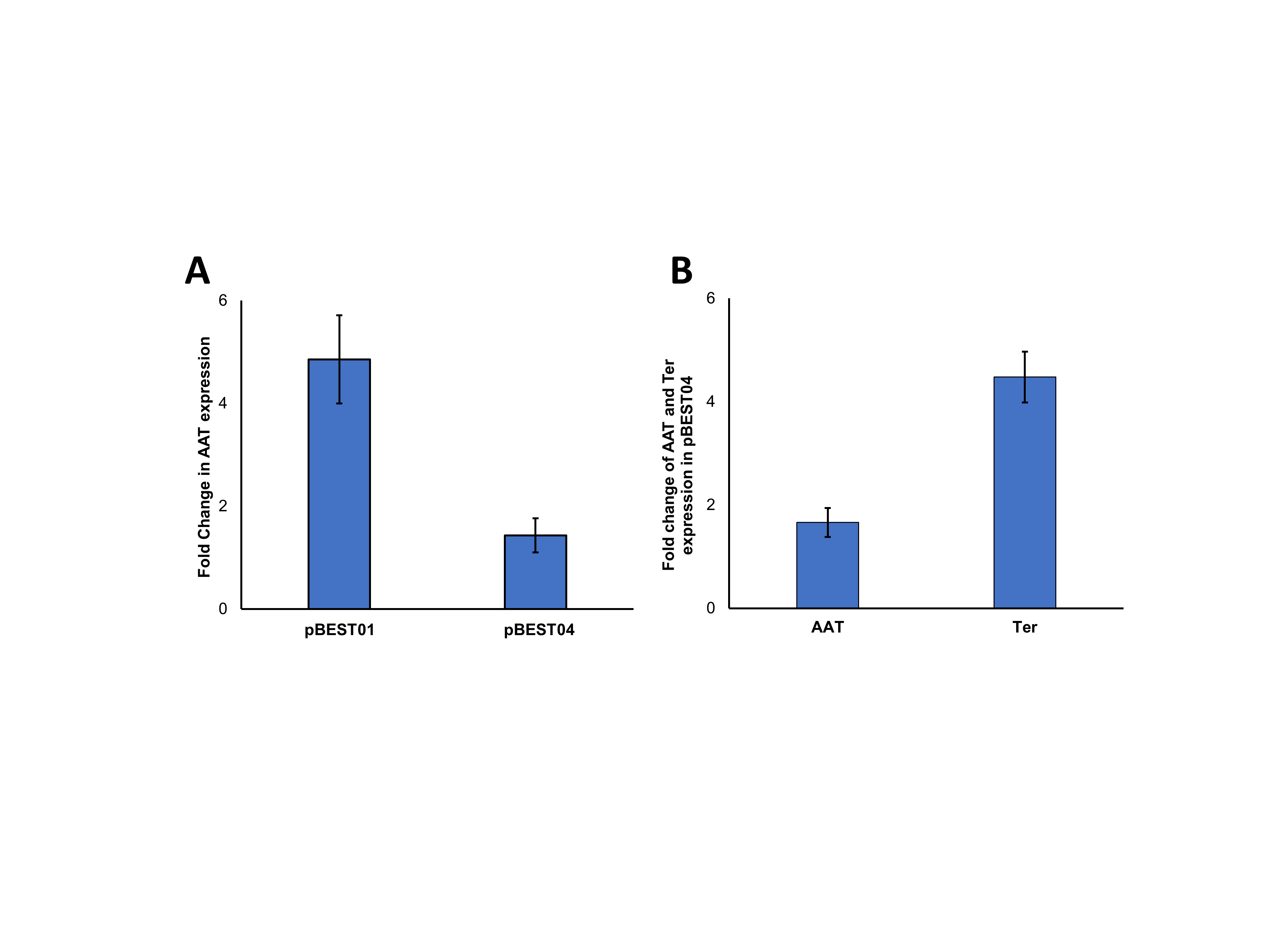

Supplement: Supplemental Information 21 — Fold change in expression of A the AAT16 gene in E. coli expressing either plasmid pBEST01 (p15A, HygBR, T7, AAT16Ac) or pBEST04 (p15A, HygBR, T7, AAT16Ac, T7, TerTd, FdhCb), B fold change in expression of either the AAT16 or the Ter gene in E. coli expressing plasmid pBEST04. Values are given as fold change in expression relative to wildtype signal–the no template control (=1.0)–using 16S ribosomal RNA (rrsA) as a reference gene. Data is the mean + standard deviation of three biological replicates. [file peerj-07-6971-s021.png]

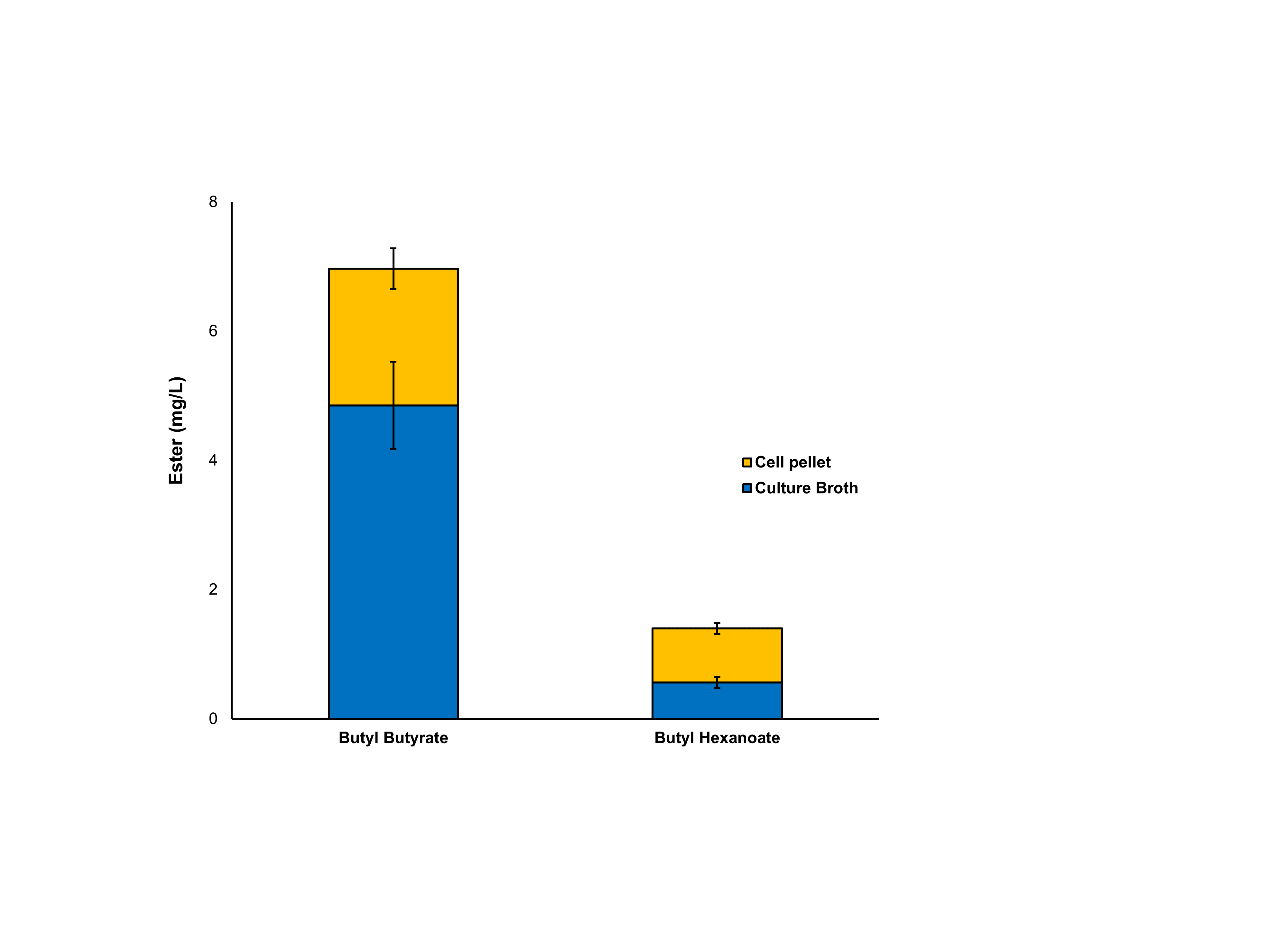

Supplement: Supplemental Information 22 — Total production of butyl butyrate and butyl hexanoate from E. coli C43 (DE3) expressing the harmonised AAT16 from A. chinensis upon exogenous addition of only 10 mM butanol. Products were analysed from both the culture supernatant and cell pellet after 18h of growth at 20°C. Data are the mean ± standard deviation from three biological replicates. [file peerj-07-6971-s022.png]

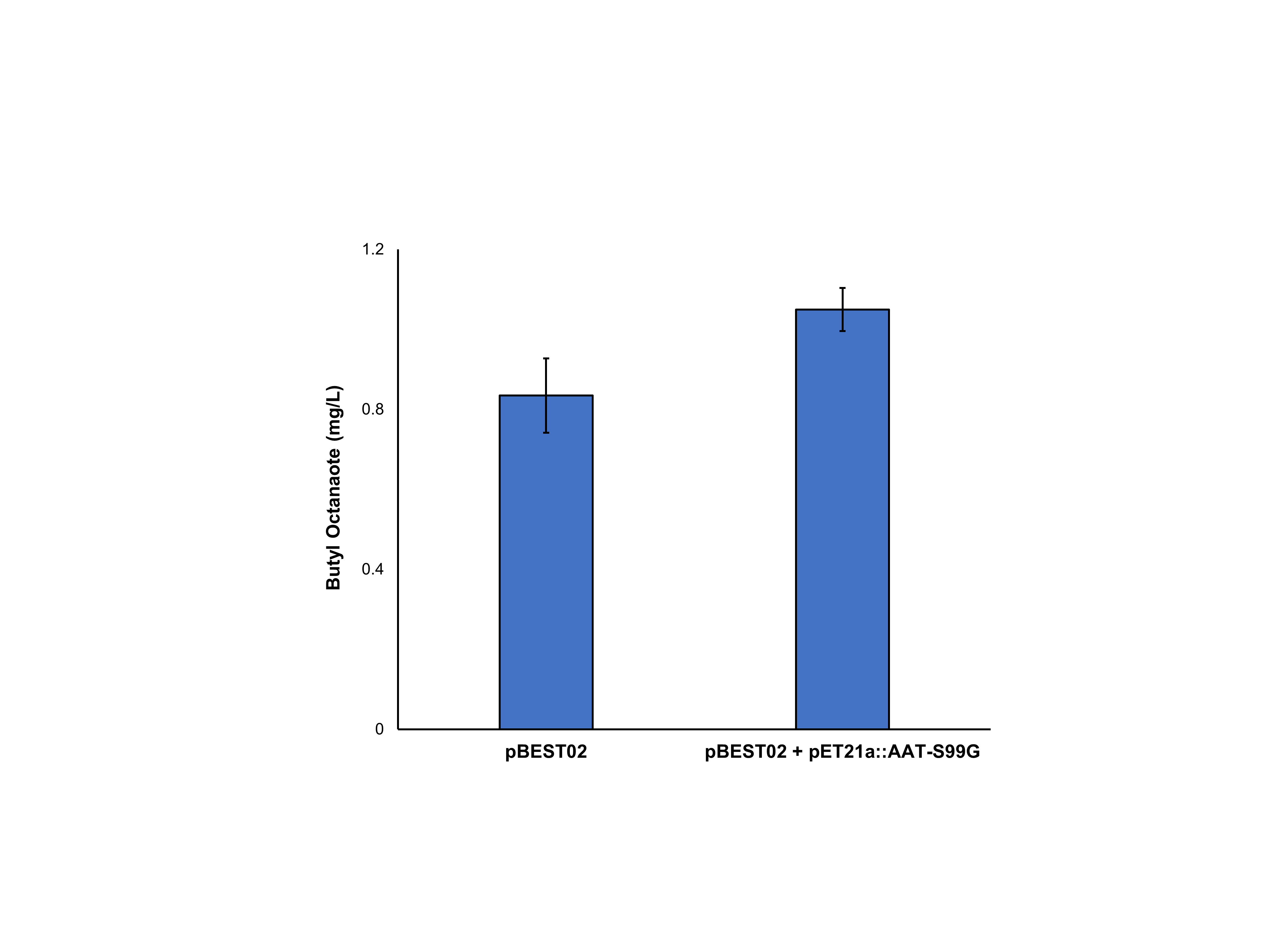

Supplement: Supplemental Information 23 — Production of butyl octanoate from E. coli C43 (DE3) harbouring either pBEST02 or pBEST02 + pET21a::AAT16-S99G upon exogenous addition of 10 mM butanol and 5 mM octanoic acid. Products were analysed from both the culture supernatant and cell pellet after 18h of growth at 20°C. Data are the mean ± standard deviation from three biological replicates. [file peerj-07-6971-s023.png]

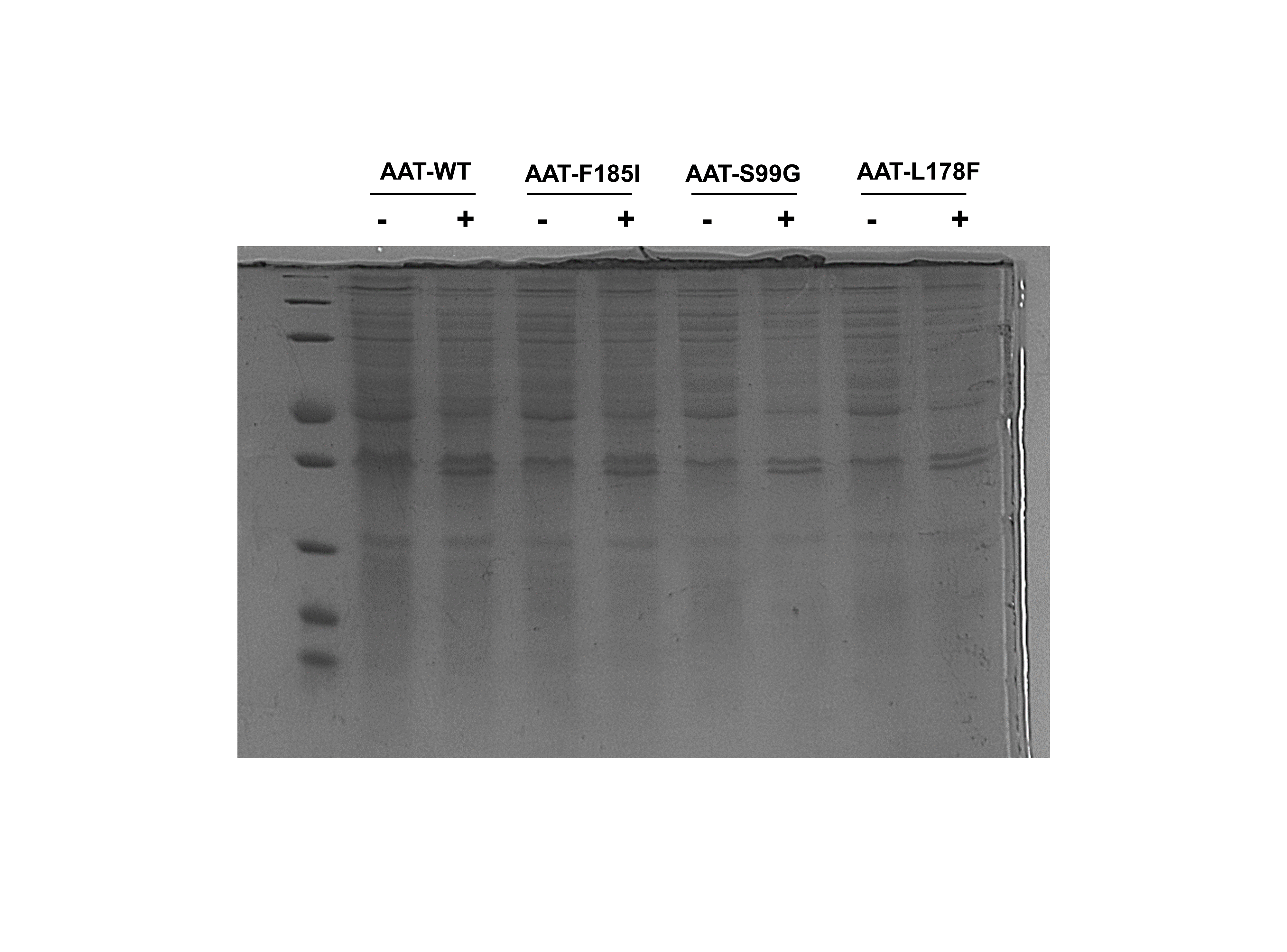

Supplement: Supplemental Information 24 — SDS-PAGE gel demonstrating the expression of AAT-wt, AAT-F185I, AAT-S99G, and AAT-L178F after 18 hours with (+) or without (-) IPTG induction. [file peerj-07-6971-s024.png]

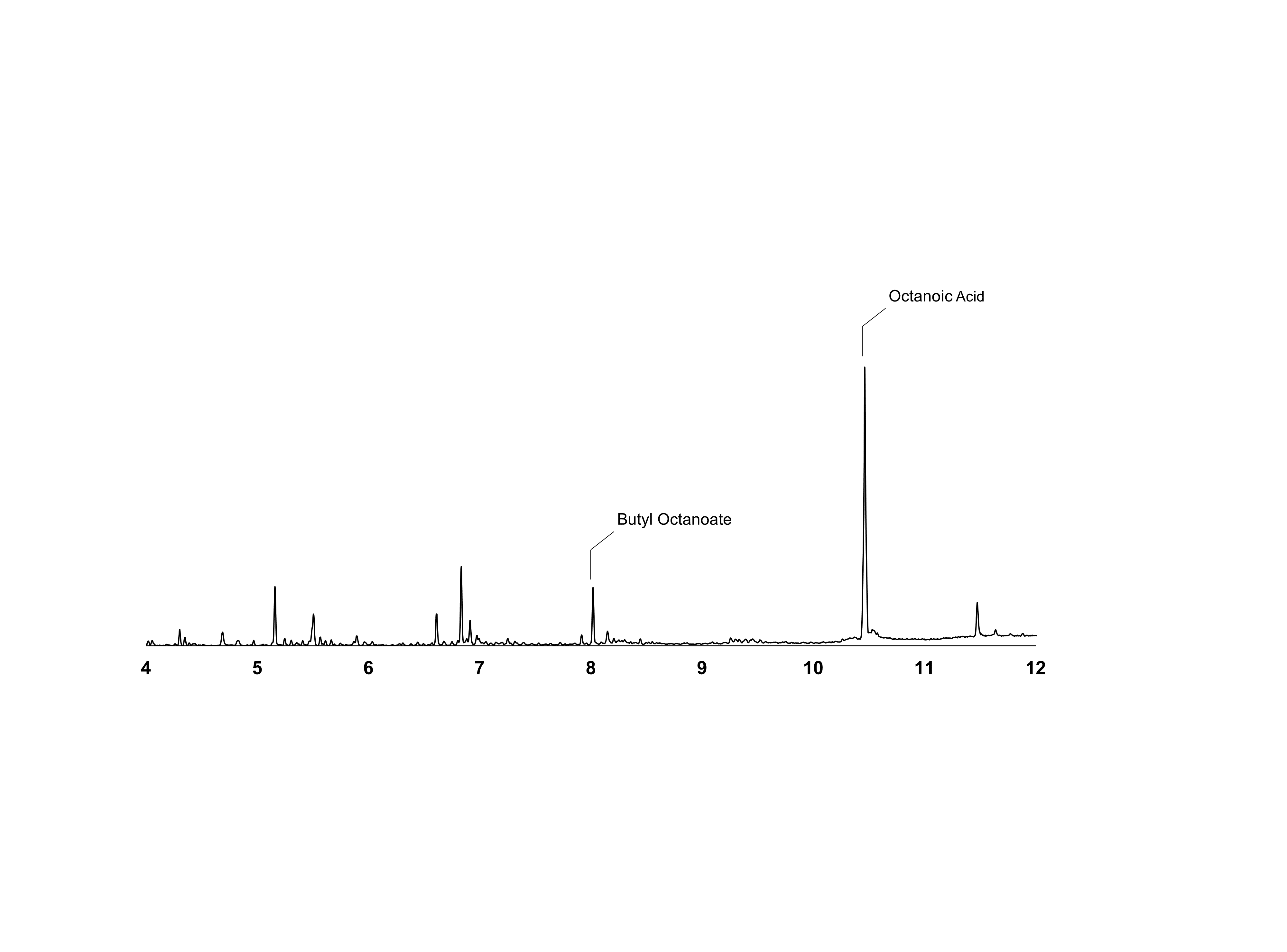

Supplement: Supplemental Information 25 — Gas chromatogram showing residual octanoic acid at time of product analysis of strain DLBO1 supplemented with 10 mM butanol and 5 mM octanoic acid. Cultures were incubated for 18h at 20°C. [file peerj-07-6971-s025.png]

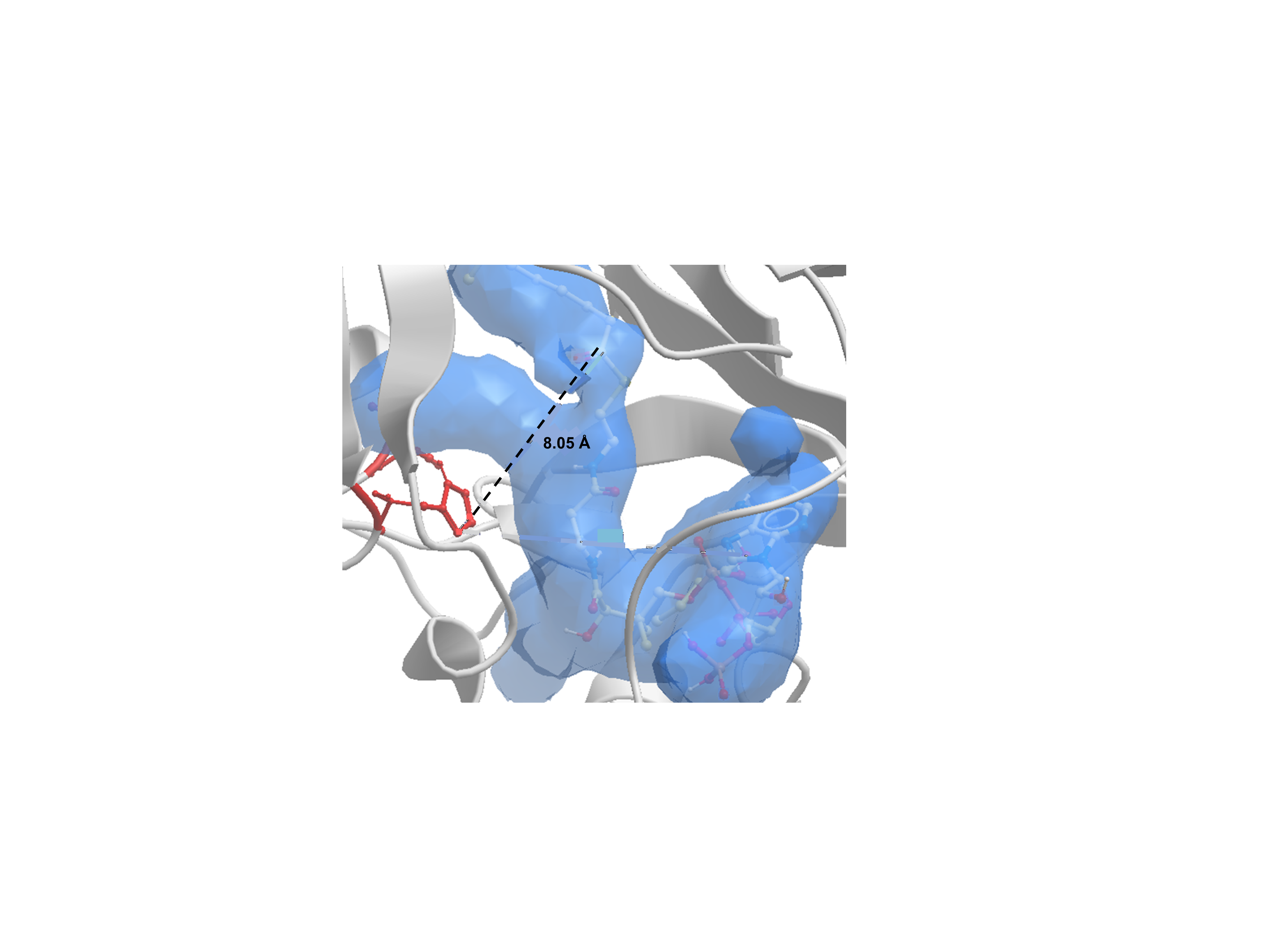

Supplement: Supplemental Information 26 — Spatial distribution of substrates butanol and octanoyl-CoA in the active site of AAT16-S99G-L178F. Distance (Å) from the ɛ nitrogen of H167 to the carbonyl group of the octanoyl-CoA and the hydroxyl group of the butanol is indicated. [file peerj-07-6971-s026.png]

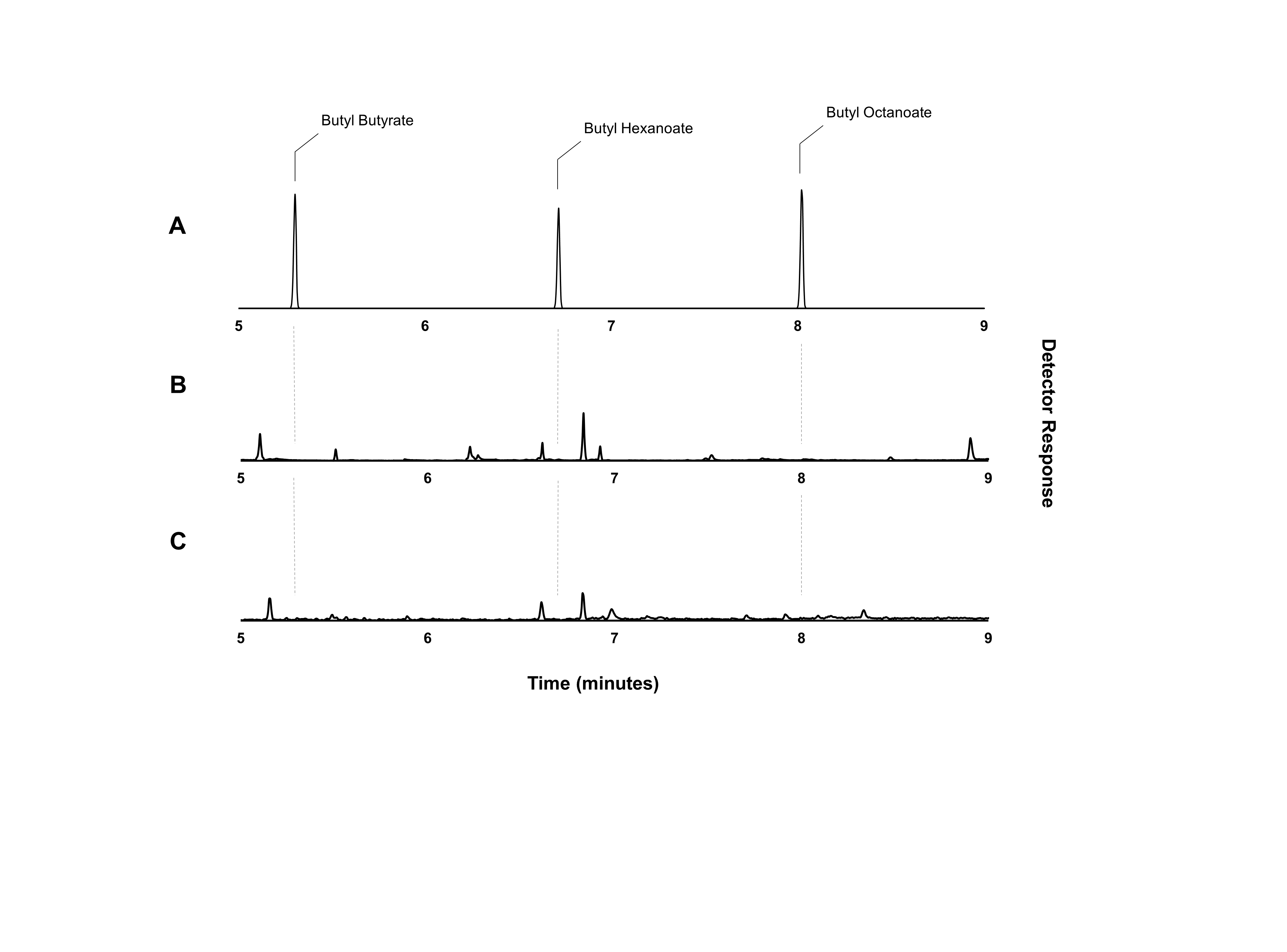

Supplement: Supplemental Information 27 — Gas chromatograms showing lack of butyl ester production from strain DLBO2 B and DLBO3 C supplemented with only 5 mM octanoic acid after 24h of growth at 20°C. Panel A shows the retention times of butyl butyrate, -hexanoate, and -octanoate standards. [file peerj-07-6971-s027.png]
