# Supplementary material for: Engineering Escherichia coli for the production of butyl octanoate from endogenous octanoyl-CoA
Source: PeerJ. 2019 Jul 1;7:e6971. doi: 10.7717/peerj.6971 (PMC6610577; doi:10.7717/peerj.6971)
Supplement: Supplemental Information 16 — Full length coding sequences of the genes used in this study. Harmonisation and optimisation was done for the genome of E. coli. [file peerj-07-6971-s016.pdf]

> Harmonised *AAT16* sequence from *A. chinensis*

ATGGCGTCTTTTCCGCCTTCGTTAGTTTTCACAGTTCGCCGCAACGAGCCTACGCTGGTGTTACCTAGCAAATC
GACGCCGCGGGAATTAAAACAGTTATCTGACATAGATGATCAGGAAGGTTTACGGTTTCAAGTGCCTGTTATC
ATGTTCTACAAGCGCAAACTGTCGATGGAAGGTGAAGACCCTGTAAAGGTAATCCGCGAAGCGTTAGCGGAA
GCGCTGGTGTTTTACTACCCGTTCGCAGGTCGCCTGATTGAGGGTCCAAATCGGAAGCTGATGGTGGATTGTA
CAAGTGAAGGGGTGTTATTCATAGAGGCGGATGCGGATATTGAGGTCAATCAACTCATTGGTGACACGATTGA
TCCGGGTTTTAGCTACCTCGACGAGCTGCTGCATGACGTGCCGGGGAGCGAAGGTATCCTGGGGTGTCCGTTA
CTGCTGATTCAAGTTACGCGCTTTCGCTGTGGTGGTTGGGCTTTTGCAATACGACTCAATCACACGATGTCTGA
TGCCCCGGGGTTAGTGCAGCTTCTTACCACCATTGCGGAGTTTGCGCGGGGGGCGGAGGGTGCGCCGAGCGTT
CCGCCCGTTTGGCAGCGCGAGTTCCTCGCGGCGCGCCAGCCGCCGTCGATCACGTTCCAACATCACGAATACG
AGCAGGTTATCAATACGACGACAGATGACAATAAGAGTATGACGCACAAAAGCTTTTTTTTCGGTCCAAAAGA
GATACGCGCAATTCGGAGTCATTTTCCGCCGCACTATCGGTCTGTAAGTTCGACCTTCGACGTATTAACGGCTT
GCCTTTGGCGCTGTCGAACCTGCGCGCTGGGGCTTGACCCTCCGAAGACAGTGCGGATCTCGTGTGCGGCAAA
TGGTCGGGGTAAACACGACTTACATGTACCGCGGGGTTACTATGGTAACGTATTTGCATTTCCTGCGGTAGTGT
CGCGCGCGGGCATGATTAGCACCAGCTCGCTGGAATACACAGTGGAGGAGGTTAAAAAGGCTAAAGCGCGCA
TGACGGGTGAGTATTTACGCTCTGTTGCGGATCTGATGGTGACGAAAGGTCGGCCGTTATATACGGTTGCGGG
GAACTATATTGTTAGCGATACAACGCGGGTGGGGTTTGACGCTATAGATTTTGGGTGGGGGAAGCCAGTATAT
GGGGGGCCTGCGCGCGCTTTCCCGCTGATTAGTTTCTATGCGCGCTTTAAGAATAATCGCGGGGAAGATGGGA
CCGTTGTATTAATTTGTCTGCCGGAAGCAGCGATGAAGCGCTTTCAGGATGAGCTGAAAAAGATGACCGAAGA
GCATGTTGACGGTCCGTTTGAATACAAATTAATTAAGGTGATGTCGAAGTTATAA

> Optimised *FATB1* sequence from *C. palustris*

ATGGTTGCAGCAGCAGCAAGCAGCGCATGTTTTCCGGTTCCGAGTCCGGGTGCAAGCCCGAAACCGGGTAAAT
TAGGTAATTGGAGCAGCAGCCTGAGTCCGAGCCTGAAACCGAAAAGCATTCCGAATGGTGGTTTTCAGGTTAA
AGCAAATGCAAGCGCACATCCGAAAGCCAATGGTAGCGCAGTTACCCTGAAAAGCGGTAGCCTGAATACCCA
AGAAGATACCCTGAGCAGCAGTCCGCCTCCGCGTGCCTTTTTTAACCAGCTGCCGGATTGGAGCATGCTGCTG
ACCGCAATTACCACCGTTTTTGTTGCACCGGAAAAACGTTGGACCATGTTTGATCGTAAAAGCAAACGTCCGA
ATATGCTGATGGATAGCTTTGGTCTGGAACGTGTTGTTCAGGATGGTCTGGTTTTTCGTCAGAGCTTTAGCATT
CGTAGCTATGAAATTTGTGCAGATCGTACCGCAAGCATTGAAACCGTTATGAATCATGTTCAAGAAACCAGCC
TGAATCAGTGCAAAAGCATTGGTCTGCTGGATGATGGTTTTGGTCGTAGTCCGGAAATGTGTAAACGTGATCT
GATTTGGGTTGTGACCCGCATGAAAATTATGGTTAATCGTTATCCGACCTGGGGTGATACCATTGAAGTTAGC
ACCTGGCTGAGCCAGAGCGGTAAAATTGGTATGGGTCGTGATTGGCTGATTAGCGATTGTAATACCGGTGAAA
TTCTGGTTCGTGCAACCAGCGTTTATGCAATGATGAATCAGAAAACCCGTCGCTTTAGCAAACTGCCGCATGA
AGTTCGTCAAGAATTTGCACCGCATTTTCTGGATAGCCCTCCGGCAATTGAAGATAATGATGGTAAACTGCAG
AAATTTGATGTCAAAACCGGTGATAGCATTCGCAAAGGTCTGACCCCTGGTTGGTATGATCTGGATGTTAATC
AGCATGTTAGCAACGTGAAATATATCGGCTGGATTCTGGAAAGCATGCCGACCGAAGTTCTGGAAACCCAAG
AACTGTGTAGTCTGACCCTGGAATATCGTCGTGAATGTGGTCGTGATAGCGTGCTGGAAAGCGTTACCAGCAT
GGATCCGAGCAAAGTTGGTGATCGTTTTCAGTATCGTCATCTGCTGCGTCTGGAAGATGGTGCAGATATTATG
AAAGGTCGTACCGAATGGCGTCCGAAAAATGCAGGCACCAATGGTGCAATTAGCACCGGTAAAACCTAA

> Optimised *FadD*-V451A sequence from *E. coli*

ATGAAAAAAGTGTGGCTGAATCGCTATCCGGCAGATGTTCCGACCGAAATTAATCCGGATCGTTATCAGAGTC
TGGTGGATATGTTTGAACAGAGCGTTGCACGTTATGCAGATCAGCCTGCCTTTGTTAATATGGGTGAAGTTATG
ACCTTTCGCAAACTGGAAGAACGTAGCCGTGCATTTGCAGCATATCTGCAGCAAGGTCTGGGTCTGAAAAAAG
GTGATCGTGTTGCCCTGATGATGCCGAATCTGCTGCAGTATCCGGTTGCACTGTTTGGTATTCTGCGTGCAGGT
ATGATTGTTGTTAATGTGAATCCGCTGTATACACCGCGTGAACTGGAACATCAGCTGAATGATAGCGGTGCAA
GCGCCATTGTTATTGTTAGCAATTTTGCACACACCCTGGAAAAAGTTGTTGATAAAACCGCAGTTCAGCATGT
GATTCTGACCCGTATGGGTGATCAGCTGAGTACCGCAAAAGGCACCGTTGTTAATTTTGTGGTGAAATACATT
AAACGCCTGGTGCCGAAATATCATCTGCCTGATGCCATTAGCTTTCGTAGTGCACTGCATAATGGTTATCGTAT
GCAGTATGTTAAACCGGAACTGGTTCCGGAAGATCTGGCATTCCTGCAGTATACAGGTGGCACCACCGGTGTT
GCAAAAGGTGCAATGCTGACCCATCGTAATATGCTGGCCAATCTGGAACAGGTTAATGCAACCTATGGTCCGC
TGCTGCATCCGGGTAAAGAACTGGTTGTTACCGCACTGCCGCTGTATCATATCTTTGCCCTGACCATTAATTGC
CTGCTGTTTATTGAATTAGGTGGTCAGAACCTGCTGATTACCAATCCGCGTGATATTCCTGGTCTGGTGAAAGA
GCTGGCAAAATATCCGTTTACCGCCATTACCGGTGTGAATACCCTGTTTAATGCACTGCTGAATAACAAAGAA
TTCCAGCAGCTGGATTTTAGCAGCCTGCATCTGAGTGCCGGTGGTGGTATGCCGGTGCAGCAGGTTGTTGCAG
AACGTTGGGTTAAACTGACAGGTCAGTATCTGCTGGAAGGTTATGGTCTGACCGAATGTGCACCGCTGGTTAG
CGTTAATCCGTATGATATTGATTATCATAGCGGTAGCATTGGCCTGCCGGTGCCGAGCACCGAAGCAAAACTG
GTGGATGATGATGATAATGAAGTTCCGCCTGGTCAGCCTGGTGAACTGTGTGTTAAAGGTCCGCAGGTTATGT
TAGGTTATTGGCAGCGTCCGGATGCAACCGATGAAATTATCAAAAATGGTTGGCTGCATACGGGTGATATTGC
AGTTATGGATGAAGAAGGTTTTCTGCGTATTGCCGATCGCAAAAAAGACATGATTCTGGTGAGCGGCTTTAAC

GTTTATCCGAATGAAATCGAAGATGTGGTTATGCAGCATCCTGGTGTTCAAGAGGTTGCAGCCGTTGGTGTTC
CGAGCGGTAGCTCAGGTGAAGCAGTTAAAATCTTCGTGGTGAAAAAAGATCCGTCACTGACCGAAGAAAGCC
TGGTTACCTTTTGTCGTCGCCAGCTGACCGGTTATAAAGTTCCGAAACTGGTTGAATTTCGTGATGAACTGCCG
AAAAGTAACGTGGGTAAAATTCTGCGTCGTGAACTGCGTGATGAAGCACGTGGTAAAGTTGATAATAAAGCC
TAA
