# Supplementary material for: Engineering Escherichia coli for the production of butyl octanoate from endogenous octanoyl-CoA
Source: PeerJ. 2019 Jul 1;7:e6971. doi: 10.7717/peerj.6971 (PMC6610577; doi:10.7717/peerj.6971)
Supplement: Supplemental Information 17 — Ramachandran plot of AAT16 protein model. The geometric evaluations required to create this plot were performed using MolProbity software (Chen et al., 2010; Lovell et al., 2003). [file peerj-07-6971-s017.pdf]

MolProbity Ramachandran analysis

AAT16_model_clean_trimmed.pdb, model 3223

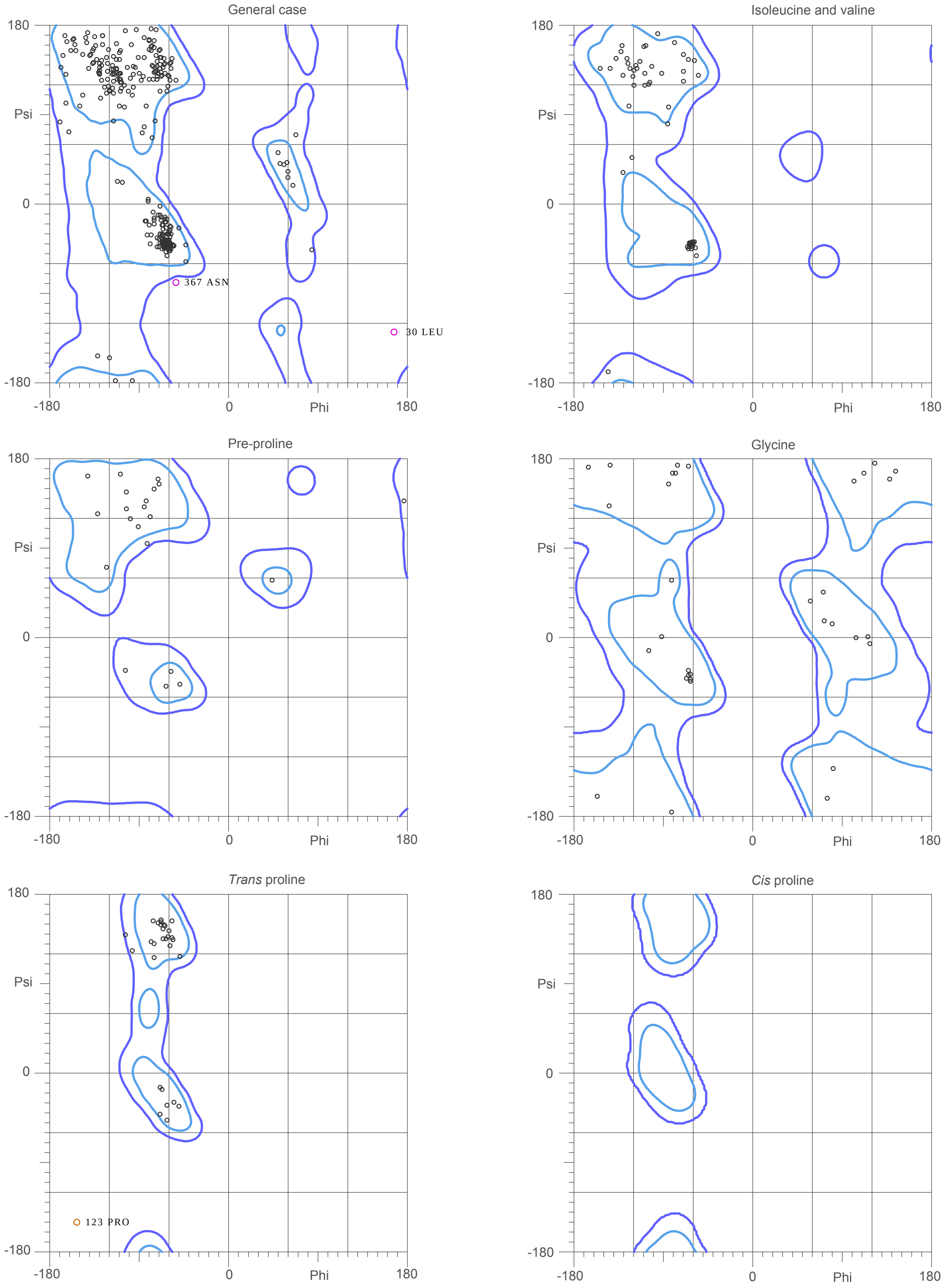

95.4% (431/452) of all residues were in favored (98%) regions.
99.3% (449/452) of all residues were in allowed (>99.8%) regions.

There were 3 outliers (phi, psi):
30 LEU (167.9, -129.2)
123 PRO (-153.6, -150.7)
367 ASN (-53.2, -80.0)

http://kinemage.biochem.duke.edu

Lovell, Davis, et al. Proteins 50:437 (2003)
