# Supplementary material for: Engineering Escherichia coli for the production of butyl octanoate from endogenous octanoyl-CoA
Source: PeerJ. 2019 Jul 1;7:e6971. doi: 10.7717/peerj.6971 (PMC6610577; doi:10.7717/peerj.6971)
Supplement: Supplemental Information 19 — Amino acid sequence alignment of fruit or flower derived AAT enzymes from a number of different plant species. Sequences correspond to Genbank accession numbers: Ae (Actinidia eriantha) AAT (HO772637); Ban (Musa sapientum) AAT (AW025506); Mp (Malus pumila) AAT (AY707098); Ac (Actinidia chinensis) AAT (HO772640); Cm (Cucumis melo) AAT1 (CAA94432), AAT2 (AAL77060), AAT3 (AAW51125), AAT4 (AWW51126); Vp (Vasconcellea pubescens) AAT (FJ548611); Cb (Clarkia breweri) BEBT (AAN09796), BEAT (AAF04787); Rh (Rosa hybrid) AAT (AAW31948); Fa (Fragaria x ananassa) SAAT (AAG13130); Fv (Fragaria vesca) VAAT (AAN07090). Sequences were aligned using Clustal Omega and BoxShade. Residues of AAT16 that were mutated are highlighted in yellow. [file peerj-07-6971-s019.pdf]

```
AeAAT      1 ----MASSV---RLVKKPVLVAPVDPTPSTVLSL-SSLDSQLFLRFPIEYLLVYASPH-G
Ban-AAT    1 --------MSFAVTRTSRSLVTPCGVTPTGSLGL-SAIDRVPGLRHMVRSLHVF------
MpAAT1     1 ----MMSFSVLQVKRLQPELITPAKSTPQETKFL-SDIDDQESLRVQIPIIMCYKDNPSL
AcAAT      1 -MASFPPSLVFTVRRNEPTLVLPSKSTPRELKQL-SDIDDQEGLRFQVPVIMFYKRKL-S
CmAAT1     1 -------DFSFHVRKCQPELIAPANPTPYEFKQL-SDVDDQQSLRLQLPFVNIYPHNP-S
CmAAT2     1 METMQTIDFSFQVRKCQPELIAPANPTPYEFKQL-SDVDDQQSLRFQLPLVNIYHHNP-S
VpAAT      1 -MAEKASSLMFNVRRHEPELITPAKPTPREIKLL-SDIDDQDGLRFQVPIIQFYKNNS-S
CmAAT3     1 ----MASSLVFQVQRSQPQLIPPSDPTPHEFKQL-SDIDDQEGLRFQIPVIQFYRHDP-R
BEBT       1 --MAHDQSLSFEVCRRKPELIRPAKQTPHEFKKL-SDVEDQEGLRFQIPVIQFYKHNNES
BEAT       1 ----------MNVTMHSKKLLKPSIPTPNHLQKLNLSLLDQIQIPFYVGLIFHYETLSDN
CmAAT4     1 ----------MEVKVLSKETIIPSSPTPPHLQPLNLSLLDQLSPMLYIPLLLFYPMKKSY
RhAAT      1 -------MEKIEVSIISRDTIKPSAAS-SSLHPYKLSIIDQFTPTTYFPVIFFYPITDRV
SAAT       1 -------MEKIEVSINSKHTIKPSTSS-TPLQPYKLTLLDQLTPPAYVPIVFFYPITDHD
VAAT       1 -------MEKIEVSIISKHTIKPSTSS-SPLQPYKLTLLDQLTPPSYVPMVFFYPITGPA

AeAAT     52 VD--RAVTAARVKAALARSLVPYYPLAGRVKTRPD-STGLDVVCQAQGAGLLEAVSDYTA
Ban-AAT   46 RQ--GREPARIIREALSKALVKYYPFAGRFVDDPEGGGEVRVACTGEGAWFVEAKADCSL
MpAAT1    56 NK--NRNPVKAIREALSRALVYYYPLAGRLREGP--NRKLVVDCNGEGILFVEASADVTL
AcAAT     58 ME--GEDPVKVIREALAEALVFYYPFAGRLIEGP--NRKLMVDCTSEGVLFIEADADIEV
CmAAT1    52 LE--GRDPVKVIKEAIGKALVFYYPLAGRLREGP--GRKLFVECTGEGILFIEADADVSL
CmAAT2    59 LE--GRDPVKVIKEAIAKALVFYYPLAGRLREGP--GRKLFVECTGEGILFIEADADVSL
VpAAT     58 MQ--GKNPAKIIKSALAETLVHYYPLAGRLREGF--GRKLMVECTGEGILFIEADADVTL
CmAAT3    55 MA--GTDPARVIKEAIAKALVFYYPFAGRLREGP--GRKLFVECTGEGVMFIEADADVSL
BEBT      58 MQ--ERDPVQVIREGIARALVYYYPFAGRLREVD--GRKLVVECTGEGVMFIEADADVTL
BEAT      51 ----SDITLSKLESSLSETLTLYYHVAGRYN-----GTDCVIECNDQGIGYVETAFDVEL
CmAAT4    51 QHQDHNKAIATLKTSLSKTLSRFYLLAGRII-----GK-S-IHCNDKGAVFMEATINSNM
RhAAT     53 FN--LPQTLTDLKNTVSQALTLYHPLSGRIK-----NN-LYIDDFEAGIPYLEARVNFHM
SAAT      53 FN--LPQTLADLRQALSETLTLYYPLSGRVK-----NN-LYIDDFEEGVPYLEARVNCDM
VAAT      53 VF--NLQTLADLRHALSETLTLYYPLSGRVK-----NN-LYIDDFEEGVPYLEARVNCDM

AeAAT    109 SDFQRA--PRSVTEWRKLLLVE-----V---FKVVPPLVVQLTWLSDGCVALGVGFSHCV
Ban-AAT  104 EDVKYLDLPL--MIPEDALLPKPCPGLN---PLDLP-LMLQVTEFVGGGFVVGLISVHTI
MpAAT1   112 EQL-GDKILPPCPLLEE-FLYNFPGSDG---IIDCPLLLIQVTCLTCGGFILALRLNHTM
AcAAT    114 NQLIGDTIDPGFSYLDE-LLHDVPGSEG---ILGCPLLLIQVTRFRCGGWAFAIRLNHTM
CmAAT1   108 EEF-WDTLPYSLSSMQNNIIHNALNSDE---VLNSPLLLIQVTRLKCGGFIFGLCFNHTM
CmAAT2   115 EQF-RDTLPYSLSSMENNIIHNSLNSDG---VLNSPLLLIQVTRLKCGGFIFGIHFDHTM
VpAAT    114 HEF-GDDLPPPFPCLVE-LLYDVPGSSG---IIDTPLLLIQVTRLKCGGFIFALRLNHTM
CmAAT3   111 EQF-GDALQPPFPCLEE-PLFDVPNSSG---VLDCPLLLIQVTRLKCGGFIFALRLNHTM
BEBT     114 EQF-GDALQPPFPCFDQ-LLFDVPGSGG---ILDSPLLLIQVTRLKCGSFIFALRLNHTM
BEAT     102 HQFLLG---EESNNLDL-LVGLSGFLSE---TETPPLAAIQLNMFKCGGLVIGAQFNHII
CmAAT4   104 FDILKE---PNNEVLTK-LLPCSLLCN-TKPIEEYPQIVVQANIFECGGIAISLCLLHKL
RhAAT    105 IDFLRL---PKIEWLNE-FVPMAPYRKETIS-EFLPLLGIQVNIFDS-GIAIGVSFSHKI
SAAT     105 TDFLRL---RKIECLNE-FVPIKPFSMEAISDERYPLLGVQVNVFDS-GIAIGVSVSHKL
VAAT     105 NDFLRL---PKIECLNE-FVPIKPFSMEAISDERYPLLGVQVNIFNS-GIAIGVSVSHKL

AeAAT    159 IDGIGSSEFLNLFAELATGRARLSEFQ-----PKPVWDRH--LLNSAGRTNL-GTH---P
Ban-AAT  158 ADGLGVVQFINAVAEIARGLP-K--PT-----VEPAWSRE--VIPN--PPKLPPGG---P
MpAAT1   167 CDAAGLLLFLTAIAEMARGAH-A--PS-----ILPVWERE--LLFARDPPRITCAH---H
AcAAT    170 SDAPGLVQLLTTIAEFARGAEGA--PS-----VPPVWQRE--FLAARQPPSITFQH---H
CmAAT1   164 ADGFGIVQFMKATAEIARGAF-A--PS-----ILPVWQRA--LLTARDPPRITFRH---Y
CmAAT2   171 ADGFGIAQFMKAIAEIARGAF-A--PS-----ILPVWQRA--LLTARDPPRITVRH---Y
VpAAT    169 SDASGLVQFMTAVGEMARGQR-S--LS-----IQPVWERH--LLNARDPPRVTHIH---H
CmAAT3   166 SDASGLVQFMMAVGEMARGAT-A--PS-----VRPVWQRA--LLNARDPPKVTCHH---R
BEBT     169 ADAAGIVLFMKAVGEMARGAA-T--PS-----TLPVWDRH--ILNARVPPQVTFNH---R
BEAT     155 GDMFTMSTFMNSWAKACRVGIK--------EVAHPTFGLAP-LMPSAK------------
CmAAT4   159 IDAATFCCFLRSWATTNRELLSLDHSSPNNNMVCVDYKSFSSLFPQTN--LLPFHQSLIN
RhAAT    159 NDGQTASCFLKSWVAIFRGYRN--------KIIHPNLSQAALLPSRD--DLPEK-----
SAAT     160 IDGGTADCFLKSWGAVFRGCRE--------NIIHPSLSEAALLFPPRD--DLPEK-----
VAAT     160 IDGRTSDCFLKSWCAVFRGSRD--------KIIHPNLSQAALLFPPRD--DLPEK-----

AeAAT    208 EFGRVPDLSGFV-TRFTQERLSPTSITFDKTWLKELKNIAMSTSQPG---E--FPYTSFE
Ban-AAT  203 P----------V---FPSFKLLHATVDLSPDHIDHVKS-----RHLE---LTGQRCSTFD
MpAAT1   214 EYEDVIGHSDGSYASSNQSNMVQRSFYFGAKEMRVLRK-----QIPP---HLISTCSTFD
AcAAT    218 EYEQVINTT--T---DDNKSMTHKSFFFGPKEIRAIRS-----HFPP---HYRSVSSTFD
CmAAT1   211 EYDQVVDMKSGL---IPVNSKIDQLFFFSQLQISTLRQ-----TLPA---HLHDCPS-FE
CmAAT2   218 EYDQVVDTKSTL---IPANNMIDRLFFFTQRQISTLRQ-----TLPA---HLHDCSS-FE
VpAAT    216 EYDDLEDTKGTI---IPLDDMVHRSFFFGPSEMAAIRR-----LVPA---HFHRS-TTSE
CmAAT3   213 EYDEVVDTKGTI---IPLDDMAHRSFFFGPSEISAIRK-----ALPS---HLRQCSS-FE
```

```
BEBT      216 EYEEVKGT--IF---TPFDDLAHRSFFFGSTEISAMRK-----QIPP---HLRSCSTTIE
BEAT      194 -VLNIPPP-----PSFEGVKFVSKRFVFHENALTRLRKEATEEDGDGDDDQKKKRPSRVD
CmAAT4    217 NDKAVVPP-----SSIFNRKRRFQRFVFRSEAILDLKAKAKSC--------DIPNPTCVE
RhAAT     204 -YVAMMER-----MWFGEKKVVTRRFVFDAKAISALQDEGKSE--------YVPKPSRVQ
SAAT      205 -YVDQMEA-----LWFAGKKVATRRFVFGVKAISSIQDEAKSE--------SVPKPSRVH
VAAT      205 -YARQMEG-----LWFVGKKVATRRFVFGAKAISVIQDEAKSE--------SVPKPSRVQ

AeAAT     262 VLSGHIWRSWARSLNLP-----AKQVLKLLFSINIRNRVK----PSLPAGYYGNAFVLGC
Ban-AAT   242 VAIANLWQSRTRAINLD-----PGVDVHVCFFANTRHLLRQVVLLPPEDGYYGNCFYPVT
MpAAT1    266 LITACLWKCRTLALNIN-----PKEAVRVSCIVNARGKHNN---VRLPLGYYGNAFAFPA
AcAAT     265 VLTACLWRCRTCALGLD-----PPKTVRISCAANGRGKHD----LHVPRGYYGNVFAFPA
CmAAT1    259 VLTAYVWRLRTIALQFK-----PEEEVRFLCVMNLRSKID----I--PLGYYGNAVVVPA
CmAAT2    266 VLAAYVWRLRTIAFQLK-----PEEEVRFLCVVNLRSKID----I--PLGFYGNAIVFPA
VpAAT     264 VLTAYLWRCYTIALQPD-----PEEEMRVICVVNSRTKLN----PPLPTGFYGNGIAFPA
CmAAT3    261 VLTACLWRFRTISLQPD-----PEEEVRVLCIVNSRSKFN----PPLPTGYYGNAFAFPV
BEBT      263 VLTACLWRCRTLAIKPN-----PDEEVRMICIVNARSKFN----PPLPDGYYGNAFAIPA
BEAT      248 LVTAFLSKSLIEMDCAPK-ELTKSRPSLMVHMMNLRKRTKL---ALE-NDVSGNFFIVVN
CmAAT4    264 TLTCFIWKYLMKVADDGD----SQRPSTLSHVVNIRKML-E---PSLGEVSLGNIMWGTV
RhAAT     250 ALTGFLWKHQLAASRALSSGT-STRFSVASQTVNLRSKMNM---KTTLDNAIGNIFLWAS
SAAT      251 AVTGFLWKHLIAASRALTSGTTSTRLSIAAQAVNLRTRMNM---ETVLDNATGNLFWWAQ
VAAT      251 AVTSFLWKHLIATSRALTSGTTSTRLSIATQVVNIRSRRNM---ETVWDNAIGNLIWFAP

AeAAT     313 AQ----TSVKDLTEKGLGYCADLVRGAKERVGDEYAREVVESVSWP-------------R
Ban-AAT   297 AT----APSGRIASAELIDVVSIIRDAKSRLPGEFAKWAAGDFKDD-------------P
MpAAT1    318 AI----SKAEPLCKNPLGYALELVKKAKATMNEEYLRSVADLLVLR-------------G
AcAAT     316 VV----SRAGMISTSSLEYTVEEVKKAKARMTGEYLRSVADLMVTK-------------G
CmAAT1    308 VI----TTAAKLCGNPLGYAVDLIRKAKAKATMEYIKSTVDLMVIK-------------G
CmAAT2    315 VI----TTVAKLCGNPLGYAVDLIRKAKAKATKEYIKSMVDFMVIK-------------G
VpAAT     315 AI----SQAKKICENPFGYTLQLVKQTKVDVTEEYMRSAADLMAMK-------------G
CmAAT3    312 AL----TTAGKLCQNPLGYALELVRKAKADVTEDYMKSVADLMVIK-------------G
BEBT      314 AV----TTAGKLCNNPLGFALELIRKAKREVTEEYMHSVADLMVAT-------------G
BEAT      303 AESK-ITVAPKITDL---------TESLGSACGEIISEVAKVDDAEVVSSMVLNSVREFY
CmAAT4    316 AHHFSTTRNEEFEGLELSKLVSLLRQSFKKINKDYIKELIMGGDKE-RRNGVMKLVGE-I
RhAAT     306 ARLDLNDTAPGSSDLKLCDLVNLLNESIKEFNSDYLEILKGKEGYG-GMCDLLDFMEEGS
SAAT      308 AILELSHTTPEISDLKLCDLVNLLNGSVKQCNGDYFETFKGKEGYG-RMCEYLDFQRTMS
VAAT      308 AILELSHTTLEISDLKLCDLVNLLNGSVKQCNGDYFETFMGKEGYG-SMCEYLDFQRTMS

AeAAT     356 RASPDSVGVLI-ISQWSRLGLDRVDFGLGRP--VQVGPICC--------DRYCLFLPVR-
Ban-AAT   340 Y-ELSFTYNSLFVSDWTRLGFLDVDYGWGKPLHVIPFAYLDIMAVGIIG------APPA-
MpAAT1    361 RPQYSSTGSYLIVSDNTRVGFGDVNFGWGQ--PVFAGPV--KA-----LDLISFYVQHKN
AcAAT     359 RPLYTVAGNYI-VSDTTRVGFDAIDFGWGK--PVYGGPA--RA-----FPLISFYARFKN
CmAAT1    351 RPYFTVVGSFM-MSDLTRIGVENVDFGWGK--AIFGGPT--TTGARITRGLVSFCVPFMN
CmAAT2    358 RPRFTEIGPFM-MSDITRIGFENVDFGWGK--AIFGGPI--IGGCGIIRGMISYSIAFMN
VpAAT     358 RPHFTVVRRYM-VSDVTRAGFGLVDFGWGRPEPVYGGPA--KGGVGPIPGVTSFFVPFKN
CmAAT3    355 RPHFTVVRTYL-VSDVTRAGFEDVDFGWGK--AMYGGPA--KGGVGAIPGVASFYIPFKN
BEBT      357 RPHFTVVNTYL-VSDVTRAGFGEVDFGWGE--AVYGGPA--KGGVGVIPGVTSFYIPLRN
BEAT      353 YEWGKGEKNVFVYSSWCRFPLYEVDFGWGIPSLV--DTT------AFP---FGLIVLMDE
CmAAT4    374 NKWP--ISNYYFFTSWKNLKLNEVDFGWGKPLWS--AIA------GDPNEMMGNIIVLVD
RhAAT     365 FVEP--APEFYSFSSWTRF-FDQVDFGWGRPSWV--GFS------GRVETR--NFTIFVE
SAAT      367 SMEP--APDIYLFSSWTNF-FNPLDFGWGRTSWI--GVA------GKIESASCKFIILVP
VAAT      367 SMEP--APEIYLFTSWTNF-FNQLDFGWGRTSWI--GVA------GKIESAFCNLTTLVP

AeAAT     404 -D-RTESVKVMVAVPTSAVDRYEYFIRSPYS-----------------------------
Ban-AAT   392 -P-QKGTRVMAQCVEKEHMQAFLEEMKGFA------------------------------
MpAAT1    412 -N-TEDGILVPMCLPSSAMERFQQELERITQEPKE-DICN-----NLRSTSQ--------
AcAAT     409 NR-GEDGTVVLICLPEAAMKRFQDELKKMTEEH--VDGPFEYKLIKVMSKL---------
CmAAT1    406 RN-GEKGTALSLCLPPPAMERFRANVHASLQVKQVVDAVDSHMQ-TIQSASK--------
CmAAT2    413 RN-GEKGIVVPLCLPPPAMERFRANVHASLQVIQVLDKVDRDMQ-TILSAL---------
VpAAT     415 RK-GEKGIVVPTCLPTPAMERFAKLMNEILQNQLLVSAE-ENKSVFIVSAI---------
CmAAT3    410 KK-GERGILVPLCLPAPAMERFVKELDALLKAGKTIDGVDNKKPLFIASAL---------
BEBT      412 RQ-GEKGIVLPICLPSAAMEIFAEALNNTLNGKE-IEIAK----HFTQSSL---------
BEAT      402 APAGDG-IAVRACLSEHDMIQFQQHHQ-------LLSYVS--------------------
CmAAT4    424 NVLDDGSTEAWILLDEKEMQLLEQIPQ-------FLEFALLNPSINLPHNQKTADEIFSN
RhAAT     412 TQCDDG-IDAWVTVDEKQMAMLEQDPQ-------FLAFASPNPRISIASSVGMD------
SAAT      416 TQCGSG-IEAWVNLEEEKMAMLEQDPH-------FLALASPKTLI---------------
VAAT      416 TPCDTG-IEAWVNLEEEKMAMLEQDPQ-------FLALASPKTLISRY------------
```

```
AeAAT        ---
Ban-AAT      ---
MpAAT1       ---
AcAAT        ---
CmAAT1       ---
CmAAT2       ---
VpAAT        ---
CmAAT3       ---
BEBT         ---
BEAT         ---
CmAAT4   477 KLI
RhAAT        ---
SAAT         ---
VAAT         ---
```
