# Supplementary material for: Engineering Escherichia coli for the production of butyl octanoate from endogenous octanoyl-CoA
Source: PeerJ. 2019 Jul 1;7:e6971. doi: 10.7717/peerj.6971 (PMC6610577; doi:10.7717/peerj.6971)
Supplement: Supplemental Information 28 — Ter assay performed at 30°C and 340 nm using 200 μmol NADH, 2 μmol FAD, and 200 μmol crotonyl-CoA. AAT16 assay performed at 30°C for 30 minutes using 10 mM butanol and 0.75 mM hexanoyl-CoA. [file peerj-07-6971-s028.docx]

|  | **Activity (µmol product/min/mg crude protein)** | |  |
| --- | --- | --- | --- |
|  | **Ter^a^** | **AAT16^b^** | |
| **C43 (DE3)** | 0.22 + 0.003 | -- | |
| **pBEST01** | 0.16 + 0.01 | 9.34x10^-3^ + 8.8x10^-4^ | |
| **pBEST04** | 2.31 + 0.14 | 1.65x10^-4^ + 1.9x10^-5^ | |

^a^ Ter assay performed at 30℃ and 340 nm using 200 µmol NADH, 2 µmol FAD, and 200 µmol crotonyl-CoA

^b^ AAT16 assay performed at 30℃ for 30 minutes using 10 mM butanol and 0.75 mM hexanoyl-CoA
