# Supplementary material for: Engineering Escherichia coli for the production of butyl octanoate from endogenous octanoyl-CoA
Source: PeerJ. 2019 Jul 1;7:e6971. doi: 10.7717/peerj.6971 (PMC6610577; doi:10.7717/peerj.6971)
Supplement: Supplemental Information 29 — In the plasmid description, subscript letters refer to the source of the gene as follows: Ac = Actinidia chinensis, Td = Treponema denticola, Cb = Candida boidinii. [file peerj-07-6971-s029.docx]

| **Name** | **Description** | **Reference** |
| --- | --- | --- |
| pBEST01 | p15A, HygB^R^, T7, *AAT16*_Ac_ | This study |
| pBEST02 | p15A, HygB^R^, T7, *AAT16-S99G*_Ac_ | This study |
| pBEST04 | p15A, HygB^R^, T7, *AAT16*_Ac_, T7, *Ter*_Td_, *Fdh*_Cb_ | This study |

In the plasmid description, subscript letters refer to the source of the gene as follows: Ac = *Actinidia chinensis,* Td = *Treponema denticola*, Cb = *Candida boidinii*
