# Supplementary material for: Engineering Escherichia coli for the production of butyl octanoate from endogenous octanoyl-CoA
Source: PeerJ. 2019 Jul 1;7:e6971. doi: 10.7717/peerj.6971 (PMC6610577; doi:10.7717/peerj.6971)
Supplement: Supplemental Information 30 — Oligonucleotide primers used in this study. [file peerj-07-6971-s030.docx]

| **Primer** | **Sequence** |
| --- | --- |
| **AAT16_BamHI_For** | AAAGGATCCGCGAGCTTTCCG |
| **AAT16_XhoI_Rev** | AAACTCGAGTAACTTCGACATCACCTT |
| **AAT16S99G_For** | GATTGTACAGGTGAAGGGGTGTTA |
| **AAT16S99G_Rev** | TAACACCCCTTCACCTGTACAATCC |
| **AAT16L178F_For** | GGTTAGTGCAGTTTCTTACCACC |
| **AATL16178F_Rev** | GGTGGTAAGAAACTGCACTAACCC |
| **FATB1_XhoI_For** | AAACTCGAGTTTGTTTAACTTTAA |
| **FATB1_NcoI_Rev** | TTTCCATGGTTAGGTTTTACCGGTGC |
| **Gibson primers** |  |
| **HygR_For** | GAGTAAACTTGGTCTGACAGTTATTCCTTTGCCCTCGGAC |
| **HygR_Rev** | GTGAGTTCAGGCTTTTTACCCATACTCTTCCTTTTCAATATT |
| **pBESTBackbone_F** | AGTTCAGGCTTTTTACCCATACTCTTCCTTTTTCAATATTATTGAAG |
| **pBESTBackbone_R** | GTCCGAGGGCAAAGGAATAACTG TCAGACCAAGTTTACT |
| **HygP15A_For** | TGAGCGCAACGCAATTAATGACCGATGCCCTGGAGAG |
| **HygP15A_Rev** | ATGCTTCAATAATATTGAAAAAGGAAGAGTATGGGTAAAAAGCTCT |
| **pET21_Backbone_F** | AGTTCAGGCTTTTTACCCAATACTCTTCCTTTTTCAATATTATTGA |
| **pET21_Backbone_R** | AGGCTCTCAAGGGCATCGGTCATTAATTGCGTTGCGCTC |
| **qRT-PCR primers** |  |
| **AAT16_For** | AGCTTTCCGCCTTCGTTAGT |
| **AAT16_Rev** | TCGACAGTTTGCGCTTGTAG |
| **Ter_For** | GTCATGGGTGGAGAGGACTG |
| **Ter_Rev** | TGCTTGGGTTCTCCTTGTTC |
| **rrsa_For** | CTCAACCTGGGAACTGCATC |
| **rrsa_Rev** | ACCTGAGCGTCAGTCTTCGT |
