# Supplementary material for: Engineering Escherichia coli for the production of butyl octanoate from endogenous octanoyl-CoA
Source: PeerJ. 2019 Jul 1;7:e6971. doi: 10.7717/peerj.6971 (PMC6610577; doi:10.7717/peerj.6971)
Supplement: Supplemental Information 31 [file peerj-07-6971-s031.pdf]

Creation of plasmid pBEST03

The creation of pBEST03 involved four steps, shown graphically in Supplementary Fig. 2; the primers used for its construction can be found in Supplementary Table 1. (I) the Hygromycin resistance gene from plasmid pAG32 was inserted in place of the Ampicillin resistance gene in plasmid pBEST::GFP using Gibson assembly, where the Hygromycin gene and the pBEST::GFP backbone – excluding the Ampicillin resistance gene – were amplified with complementary overhangs before assembly. This plasmid was named pBEST::GFP+Hygromycin. (II) The Hygromycin gene and the p15A origin of replication (ori) region of pBEST::GFP+Hygromycin were then inserted in place of the Ampicillin resistance gene and the pColE1 ori region of pET21a::AAT16-S99G using Gibson assembly where each respective fragment was amplified with complementary overhangs before assembly. The amplified fragment of pET21a::AAT16-S99G did not include the basis of mobility (bom) region or the rop protein present on the pET21a plasmid. The resulting vector was named pBEST02. (III) A second operon was inserted downstream of the AAT16-S99G gene in pBEST02 by Gibson assembly. pBEST02 was cut at the *Xho*I restriction site found between the stop codon of AAT16-S99G and the 6xHis-Tag present on the plasmid. A synthesised fragment that contained from 5' to 3': 40 bp complementary upstream sequence to the pBEST02 plasmid, a T7 terminator, a T7 promoter, the *FATB1* gene, the *FadD*-V451A gene, and 40 bp complementary downstream sequence to the pBEST02 plasmid, was then assembled into pBEST02 to create pBEST03 (p15A, $HygB^R$, T7, $AAT16\text{-}S99G_{\mathrm{Ac}}$, T7, $FATB1_{\mathrm{Cp}}$, $FadD\text{-}V451A_{\mathrm{Ec}}$).

Creation of plasmid pBEST04

As described above for plasmid pBEST03, with the exception that in step (II) the Hygromycin gene and the p15A origin of replication region of pBEST::GFP+Hygromycin was inserted in the place of the of the Ampicillin resistance gene and the pColE1 ori region of pET21a::AAT16 (as opposed to pET21a::AAT16-S99G) using Gibson assembly. The resulting vector was named pBEST01. Then, in step (III) the operon that was inserted downstream of the AAT gene in pBEST01 by Gibson assembly was, from 5' to 3': 40 bp complementary upstream sequence to the pBEST01 plasmid, a T7 terminator, a T7 promoter, the *Ter* gene from *Treponema denticola*, the *Fdh* gene from *Candida boidinii*, and 40 bp downstream sequence, complementary to the pBEST01 plasmid. The resulting plasmid was called pBEST04 (p15A, $HygB^R$, T7, $AAT16_{\mathrm{Ac}}$, T7, Ter_{Td}, Fdh_{Cb}).

Assay for Trans-2-enoyl-CoA (Ter) activity

Ter activity was determined on a Cary 50 Bio spectrophotometer (Varian, UK) by measuring the decrease in absorbance at 340 nm. Reactions were carried out in a total volume of 1 mL containing: phosphate buffer pH 6.2, 200 µM NADH, 2 µl FAD, 200 µM crotonyl-CoA, and 5 µl of crude cell lysate of *E. coli* strain C43 (DE3) expressing the Ter enzyme. All ingredients, except for the cell lysate,

were pre-incubated at 30°C for 5 minutes before the addition of the lysate and the commencement of absorbance reading. Initial reaction rates were calculated from the slope of the linear potion of the curve, usually the first 60 seconds.

Purification of RNA from cell cultures and cDNA synthesis

Overnight cultures of *E. coli* strains harbouring either no plasmid, pBEST01 or pBEST04 were inoculated to 1% in TB + 20 g/L glucose and grown at 37°C and 250 rpm until on OD_{600} of 1.0 was reached. Cultures were then induced with 0.4 mM IPTG and incubated at 37°C, 250rpm for a further 16 hours. RNA was isolated from 1 mL culture samples using the GenElute™ Total RNA purification Kit (Sigma-Aldrich, UK) according to the manufacturer's instructions for the preparation of RNA from bacteria, with an extended DNA removal step of 1.5 hours. RNA concentration and purity was determined using a NanoVue Plus spectrophotometer (GE Healthcare, UK).

cDNA was synthesised from samples of 1µg RNA diluted in 10µl of nuclease free water using the Applied Biosystems High Capacity cDNA Reverse Transcription Kit (Thermo Fisher, UK).

Quantitative PCR of cDNA samples (RT-qPCR)

Primers for qPCR of the *AAT16* and *Ter* transcripts were designed using Primer3 (Koressaar *et al.*, 2007; Untergasser *et al.*, 2012) and are listed in Supplementary Table 3. Optimal primer pairs were chosen based on their amplification efficiency, determined by running qPCR on a 10-fold dilution series of cDNA template for each primer set. Standard curves of cycle threshold values (C_t) for each primer set were established from the dilution series, and primer pair efficiency was calculated based on the method described by Pfaffl (2001).

All qPCR reactions were set up in triplicate in 20µl final volumes containing 5µl of cDNA diluted 1/10 in nuclease free water, 0.25 µM forward and reverse primers, and 1X LuminoCt® SYBR® Green qPCR ReadyMix™ (Sigma-Aldrich, UK). Reactions were run using a DNAEngine Peltier Thermal Cycler coupled with a Chromo4TM Real-Time PCR Detector (BioRad, UK), and data was analysed by the Opticon 3 thermal cycler software program (BioRad, UK). The reaction conditions recommended for LuminoCt® SYBR® Green qPCR ReadyMix™ were used and specified as follows: an initial denaturation step of 95°C for 20 seconds, followed by 40 cycles consisting of a denaturation step of 95°C for 3 seconds, and an annealing/extension step of 60°C for 30 seconds. A melt curve between 55°C to 95°C of 0.2°C increments was performed for each sample to confirm primer specificity. C_t values were recorded for each sample and relative quantification in fold change in expression was calculated based on the method described by Pfaffl (2001). qPCR data was normalised between samples using the expression of the 16S ribosomal RNA gene *rrsa* as a reference.
